# A Narrative Review of Recent Antibiotic Prescribing Practices in Ambulatory Care in Tanzania: Findings and Implications

**DOI:** 10.3390/medicina59122195

**Published:** 2023-12-18

**Authors:** Amos Massele, Anastasia Martin Rogers, Deogratias Gabriel, Ashura Mayanda, Sarah Magoma, Aislinn Cook, Audrey Chigome, Giulia Lorenzetti, Johanna C. Meyer, Catrin E. Moore, Brian Godman, Omary Minzi

**Affiliations:** 1 Department of Clinical Pharmacology and Therapeutics, Hubert Kairuki Memorial University, 70 Chwaku Road Mikocheni, Dar Es Salaam P.O. Box 65300, Tanzania; 2 Department of Microbiology and Parasitology, Faculty of Medicine, Hubert Kairuki Memorial University, 70 Chwaku Road Mikocheni, Dar Es Salaam P.O. Box 65300, Tanzania; anastasia.rogers@hkmu.ac.tz (A.M.R.); deogratias.gabriel@hkmu.ac.tz (D.G.); ashura.mayanda@hkmu.ac.tz (A.M.); 3 Department of Infectious Diseases, Faculty of Medicine, University of Dodoma, Dodoma P.O. Box 582, Tanzania; sarahmagoma.sm@gmail.com; 4 Centre for Neonatal and Paediatric Infection, Institute for Infection and Immunity, St. George’s University of London, London SW17 0RE, UK; aicook@sgul.ac.uk (A.C.); glorenze@sgul.ac.uk (G.L.); camoore@sgul.ac.uk (C.E.M.); 5 Health Economics Research Centre, Nuffield Department of Population Health, University of Oxford, Oxford OX1 2JD, UK; 6 Department of Public Health Pharmacy and Management, School of Pharmacy, Sefako Makgatho Health Sciences University, Ga-Rankuwa 0208, South Africahannelie.meyer@smu.ac.za (J.C.M.); 7 South African Vaccination and Immunisation Centre, Sefako Makgatho Health Sciences University, Ga-Rankuwa 0208, South Africa; 8 Strathclyde Institute of Pharmacy and Biomedical Sciences, University of Strathclyde, Glasgow G4 0RE, UK; 9 Department of Clinical Pharmacy and Pharmacology, School of Pharmacy, Muhimbili University of Health and Allied Sciences, United Nations Rd, Dar Es Salaam P.O. Box 65013, Tanzania; minziobejayesu@gmail.com

**Keywords:** antibiotic prescribing practices, ambulatory care, antimicrobial resistance, antimicrobial stewardship programs, AWaRe classification, national action plans, quality indicators, Tanzania

## Abstract

*Background and objectives*: There are concerns with the current prescribing practices of antibiotics in ambulatory care in Tanzania, including both the public and private sectors. These concerns need to be addressed as part of the national action plan (NAP) of Tanzania to reduce rising antimicrobial resistance (AMR) rates. Issues and concerns include high rates of prescribing of antibiotics for essentially self-limiting conditions. Consequently, there is a need to address this. As a result, the aims of this narrative review were to comprehensively summarize antibiotic utilization patterns particularly in ambulatory care and their rationale in Tanzania and to suggest ways forward to improve future prescribing practices. *Materials and Methods*: We undertook a narrative review of recently published studies and subsequently documented potential activities to improve future prescribing practices. Potential activities included instigating quality indicators and antimicrobial stewardship programs (ASPs). *Results*: Published studies have shown that antibiotics are being excessively prescribed in ambulatory care in Tanzania, in up to 95% to 96.3% of presenting cases depending on the sector. This is despite concerns with their appropriateness. High rates of antibiotic prescribing are not helped by variable adherence to current treatment guidelines. There have also been concerns with extensive prescribing of ‘Watch’ antibiotics in the private sector. Overall, the majority of antibiotics prescribed across the sectors, albeit inappropriately, were typically from the ‘Access’ group of antibiotics in the AWaRe (Access/Watch/Reserve) classification rather than ‘Watch’ antibiotics to limit AMR. The inappropriate prescribing of antibiotics in ambulatory care is linked to current knowledge regarding antibiotics, AMR, and ASPs among both prescribers and patients. Recommended activities for the future include improved education for all groups, the instigation of updated quality indicators, and the regular monitoring of prescribing practices against agreed-upon guidelines and indicators. Education for healthcare professionals on ASPs should start at undergraduate level and continue post qualification. Community advocacy on the rational use of antibiotics should also include social media activities to dispel misinformation. *Conclusion*: The quality of current prescribing practices of antibiotics in ambulatory care is sub-optimal in Tanzania. This needs to be urgently addressed.

## 1. Introduction

Antimicrobial resistance (AMR) is an increasing public health concern, with some authors suggesting that AMR could be the next pandemic unless actively addressed through a range of initiatives and measures [1]. In 2019, it was estimated that, globally, there were 1.27 million deaths directly attributable to bacterial AMR, with potentially up to 4.95 million deaths associated with bacterial AMR [2]. AMR is also associated with considerable morbidity and costs [3,4,5,6,7]. The World Bank estimated that the costs of AMR could be as high as USD 3.4 trillion annually unless addressed, which is equivalent to 3.8% of the annual global gross domestic product [4]. A number of national and international initiatives have now taken place to try and reduce AMR. International initiatives include the World Health Organization’s (WHO) Global Action Plan (GAP) [8], which has translated into national action plans (NAPs) (Table S1) [9,10]. There are also regional initiatives across Africa to improve the surveillance of infectious diseases and resistance patterns as well as to develop guidelines [11,12,13,14,15]. These combined activities are critical in sub-Saharan Africa, which currently has the highest burden of AMR globally [2,16].

However, African countries are at different stages of the introduction and monitoring of their NAPs due to multiple challenges [17,18]. Challenges within sub-Saharan Africa include limited resources and personnel, including champions, to drive forward agreed-upon activities [9,17,19,20].

Recently, the WHO has reclassified antibiotics into a proposed AWaRe classification (Access, Watch, and Reserve), as well as launched the AWaRe book, which includes management suggestions for 26 common or severe clinical syndromes to reduce AMR [21,22,23]. The AWaRe book, with its classification of antibiotics, takes into account the impact of different antibiotics and their resistance potential to reduce AMR [21,22,24]. According to this system, antibiotics in the ‘Watch’ group should be carefully considered before being prescribed, whilst those in the ‘Reserve’ group should only be prescribed as a last resort in hospitals and prioritized for antimicrobial stewardship programs (ASPs) [21,23,25].

In recent years, various national and other groups have been active in Tanzania to improve the utilization of antibiotics (Table 1) and to reduce AMR rates given rising concerns regarding AMR in both hospital and ambulatory care in Tanzania (Table 2) [26]. At one stage, it was estimated that by the end of 2022, total antibiotic utilization in Tanzania would be 13 times higher than that in 2010, an appreciable proportion of which was likely to be inappropriate [27]. Encouragingly, recent studies have suggested an appreciable decrease in national antimicrobial utilization rates in the human sector in Tanzania between 2017 and 2019 influenced by ongoing activities (Table 1) [26,28].

There are also concerns currently with the suboptimal registration of essential antimicrobials in Tanzania, alongside the excessive registration of non-essential antimicrobials [54]. In addition, concerns with the availability of unnecessary fixed-dose combinations (FDCs) of antibiotics in the Essential Medicines List in Tanzania, which increases the risk of side effects and AMR [10,55]. These FDCs include the ampicillin–cloxacillin, flucloxacillin–amoxicillin, and ceftriaxone–sulbactam combinations [55]. Both these areas need addressing going forward. However, initially, a critical area for all key stakeholders in Tanzania to concentrate on in order to improve antibiotic use, thereby reducing AMR, is ambulatory care. This is because ambulatory care can account for up to 90–95% of total human antibiotic use in countries, especially in low- and middle-income countries (LMICs) [22,56,57,58]. In addition, there have been increasing concerns regarding the irrational prescribing of antibiotics among healthcare professionals (HCPs) in Tanzania, including for the management of diarrhea and respiratory illnesses in children, enhancing AMR [59,60,61,62,63]. AMR is further exacerbated in Tanzania by antibiotics being widely available and used without a prescription (Table S2) [59,64,65,66,67,68,69], enhanced by issues of affordability and convenience [59]. For instance, in their study among 59 LMICs, Hossain et al. (2023) found that only 22.4% of children in Tanzania with either a cough or fever received antibiotics from qualified sources, e.g., recognized hospitals and clinics [70]. Alongside this, the appreciable availability of left-over antibiotics in households, coupled with their ease of availability in community pharmacies and drug stores, has resulted in delays in parents seeking professional help to treat young children with community acquired pneumonia (CAP) [71]. In addition, there has been mass drug administration of azithromycin for trachoma control in Tanzania, potentially impacting on AMR [72,73]. These concerns and issues are enhanced by the appreciable prevalence of acute respiratory illnesses (ARIs) among children under five being treated at health facilities in Tanzania (85%) [74], as well as the promotional activities of pharmaceutical companies [75,76].

These issues and concerns are important, with Fink et al. (2020) finding that the mean number of antibiotics prescribed per sick child visiting a healthcare facility in Tanzania was 0·69 [77], similar to the overall total among eight participating LMICs, including six from Africa [77]. In addition, there was a high rate of prescribing of antibiotics in these children at 61% of attending patients, which is higher than the findings in Rwanda and Congo (58% each) as well as Gabon (50.0%) [74]. Having said this, in their recent systematic review, Acam et al. (2023) found that the extent of antimicrobial utilization in Tanzania, at 40% of encounters, was lower than those in Ethiopia (63%), Sudan (62%), and Kenya (54%) [78].

ASPs have been successfully introduced across LMICs, including among African countries, to improve future antimicrobial prescribing practices. This is despite the many challenges and concerns including necessary financial resources and personnel [79,80,81,82,83,84,85,86,87]. These include ASPs being introduced in Tanzania to improve future antibiotic prescribing practices [88,89], typically starting with a situational analysis [90]. ASPs instigated to date in Tanzania include assessing point-of-care testing as well as developing algorithms to improve antibiotic prescribing practices in ambulatory care [91,92,93]. This is because limited sensitivity testing is currently being performed outside of hospitals in Tanzania.

Other ongoing activities to improve antibiotic use in Tanzania include general strengthening of antimicrobial stewardship (AMS) in the country, building on the Medicines, Technologies, and Pharmaceutical Services (MtaPS) program [40,41], with strengthening ASPs in hospitals being a key element of the 2023 to 2028 NAPs [26] (Table 1). Apps have also been developed to improve the awareness of AMS among HCPs [88].

In view of the ongoing activities to improve antibiotic prescribing practices in ambulatory care in Tanzania (Table 1), alongside ongoing challenges and concerns including high AMR rates (Table 2), there is an urgent need to consolidate current knowledge regarding antibiotic prescribing practices in ambulatory care to provide future direction. This includes both the public and private ambulatory care sectors as there can be appreciable differences in prescribing habits between the sectors. In Botswana, an appreciable number of patients with upper respiratory tract infections (URTIs) are being treated by private physicians, often inappropriately with antibiotics (72.9% of patients) [94]. This is very different to patients being treated in the public system in Botswana, where in addition to patients presenting with coughs, an appreciable number also presented with sexually transmitted infections and vaginal discharges. This resulted in the appreciable prescribing of metronidazole among public HCPs in Botswana, which was very different from that in the private sector [94,95]. In Iran, the same physicians treated patients differently depending on whether they were seen in public versus private sector clinics [96]. Differences between the sectors have also been seen in Tanzania, with the greatest consumption of antimicrobials currently in the private versus public sectors [27,28]. In their study, Kamuhabwa et al. (2015) found that textbooks and the internet were the principal sources of prescribing information for physicians in the public sector, whilst in private healthcare facilities, physicians were more concerned with the proven effectiveness of prescribed medicines [97]. Physicians in the public sector were also more concerned with the costs of medicines as well as their availability compared with those in the private sector [97]. Private health facilities may also be more profit-oriented than public facilities [75], with the potential for providing unnecessary care [98]. Irunde et al. (2017) also found greater rationality in the prescribing practices of medicines in public versus private healthcare facilities [61]. The recent government’s star rating facility quality assessment program, which included both public and private healthcare facilities (Table 1), documented appreciable improvements in both sectors [37,38,99]. However, private for-profit ownership was associated with a 29% lower probability of a lesser improvement versus public facilities [37].

We are also aware that there can be concerns with the availability and distribution of public healthcare facilities especially in rural areas in Tanzania [100]. Alongside this, the current use of prescribing and quality indicators also needs to be comprehensively documented as these can form part of future quality improvement programs including ASPs, especially with ASPs promoted in the updated NAP [26]. ASPs in ambulatory care are likely to increasingly include improved diagnostic tools to reduce inappropriate prescribing of antibiotics in ambulatory care [101].

The principal objectives of this paper are, firstly, to document current prescribing patterns for antibiotics in ambulatory care and their rationale; secondly, to determine key activities to improve future prescribing practices including ongoing prescribing indicators and ASPs; and thirdly, to use the consolidated findings to suggest future activities that can be undertaken by all key stakeholder groups in the short to medium term to improve future antibiotic prescribing in ambulatory care in Tanzania thereby reducing AMR. This includes any research activities as part of ongoing developments to enhance implementation research in Tanzania [102]. We have concentrated on ambulatory care prescribing versus in-patient prescribing since, as mentioned, up to 95% of total antibiotic utilization in humans in LMICs occurs in ambulatory care [56]. Consequently, it is a critical element of a one-health approach to reducing AMR in Tanzania.

## 2. Materials and Methods

### 2.1. Our Approach and Key Questions

We principally used a narrative review to address the objectives of this paper. This review involved answering the following questions with the ultimate aim of reducing AMR in Tanzania through the improved prescribing of antibiotics in ambulatory care [83,103,104,105]:

- What have been the prescribing patterns of antibiotics in public ambulatory care settings in Tanzania in recent years?
- What have been the prescribing patterns of antibiotics in the ambulatory care private sector in Tanzania in recent years?
- What is the knowledge, attitude, and practices (KAP) towards antibiotics, AMR, and ASPs among key stakeholder groups involved in ambulatory care prescribing practices in Tanzania in recent years?
- What prescribing and quality indicators have been used in ambulatory care settings across the sectors in Tanzania in recent years to improve the appropriateness of prescribing practices?
- What ASPs, including their impact, have been instigated in ambulatory care in Tanzania in recent years to improve future antibiotic prescribing practices? In addition, what guidance can other LMICs provide to key stakeholder groups in Tanzania through their activities?
- What potential activities could be instigated by key stakeholder groups in Tanzania, including the Ministry of Health, health insurance groups, physician groups, universities, and patient groups, in the short to medium terms to improve the appropriateness of antibiotic prescribing practices across the ambulatory care sectors in Tanzania to reduce AMR?

A narrative review approach was identified as the most appropriate approach to achieve the objectives of this paper. The motivation being that this approach allows for a broader scope compared to a systematic review as a number of potential papers may not be listed in PubMed or Web of Science; however, they can provide useful insights into current activities in Tanzania. In addition, pertinent information contained within a paper may be part of a wider paper, which could be missed during a systematic review. This includes knowledge of antibiotics and AMR among patients seeking help from community pharmacies without seeking any help from HCPs in ambulatory care facilities.

We were also aware that there have been a number of reviews of various factors influencing the prescription of antibiotics in ambulatory care settings in recent years including Tanzania and beyond. In addition, there have been reviews discussing potential activities to reduce the inappropriate prescribing of antibiotics in ambulatory care. However, most studies have typically focused on higher-income countries where resources and personnel can be very different [77,78,79,83,104,106,107,108,109]. Alongside this, typically only focused on one key area such as prescribing practices in ambulatory care clinics or ASPs. As a result, making it challenging to bring together all the key aspects associated with improving the appropriateness of antibiotic prescribing practices in ambulatory care settings into one comprehensive review. This was the philosophy behind this approach.

A narrative review also gives more flexibility and greater coverage of the relevant literature to provide future direction to all key stakeholder groups, similar to our recent paper in South Africa [110]. We are aware though that there are limitations with this approach in terms of rigor. However, to minimize bias, as well as to help ensure all relevant information is included in this narrative review, the participating co-authors have considerable experience in Tanzania, across Africa and beyond, in terms of research and practice surrounding the prescribing of antibiotics in ambulatory care as well as implementing policies to improve appropriate prescribing practices. This is seen as particularly important when recommending future activities to improve the prescribing of antibiotics in Tanzanian ambulatory care, which is a key objective of the paper.

We adopted a similar approach in South Africa and other LMICs when documenting and suggesting activities to improve the care of patients with both infectious and non-infectious diseases. Alongside this, documenting challenges with implementing NAPs and ASPs across sectors in Africa to reduce AMR and potential ways to address these [17,84,111,112,113,114,115]. Consequently, we were confident that this would be an appropriate approach for this comprehensive review.

### 2.2. Search Strategy and Inclusion Criteria

A literature search was performed to address the six identified questions using a number of databases including PubMed/MEDLINE, Web of Science, and Google Scholar. In addition, we conducted a manual search of the grey literature, which included key Ministry of Health documents including any NAPs.

The search strategy, which was used to address the identified questions, included a number of search terms. The search terms were ambulatory care; antibiotics; antibiotic prescribing; antimicrobials; antimicrobial prescribing; antimicrobial stewardship; antimicrobial stewardship programs; guidelines; low- and middle-income countries; prescribing indicators; quality indicators; private healthcare facilities; public healthcare centers; and Tanzania.

In view of the possible scarcity of published literature with respect to the six key questions identified, the qualifying criteria were purposefully broad. However, only English language papers were sourced as English is the recognized international scientific language [110].

We also only concentrated on ambulatory care settings as this is where the majority of patients with infections such as URTIs are treated if they visit healthcare facilities across Africa, including Tanzania. In addition, this approach acknowledges the increasing adoption of digital technologies in ambulatory care in Tanzania as well as ongoing moves to improve ambulatory care [35,93] (Table 1).

Alongside this, we also only concentrated on documented studies from 2016 onwards to reflect improvements in digitalization and other aspects of care to improve the management of patients with infectious diseases in Tanzania in recent years (Table 1) [35]. We are aware that some research was conducted appreciably earlier than 2016 but only reported recently, e.g., the study of Wiedenmayer et al. was conducted in 2012; however, only published in 2021 [116]. Alongside this, some systematic reviews including Tanzania published in recent years included studies published in 1993, which is a concern when attempting to analyze current prescribing practices and their implications [78]. However, we were cognizant of this when making suggestions for the future. We were also aware of the excessive prescribing of antibiotics in patients with COVID-19 since the start of the pandemic among LMICs, including Tanzania, despite limited evidence of secondary infections or bacterial co-infections [117,118,119,120,121,122,123]. This has been exacerbated in Tanzania by poor adherence to infection, prevention, and control (IPC) compliance to measures surrounding COVID-19 among healthcare workers in ambulatory care [124]. In addition, antibiotics were being included in national treatment guidelines across Africa despite COVID-19 being a viral infection [125]. We wanted to reflect whether this is still the case in sourced publications.

Achieving appropriate antibiotic prescribing practices in ambulatory care settings is a critical part of achieving the goals of the NAP to reduce AMR in Tanzania. Consequently, it is important that possible interventions are initially prioritized in ambulatory care, as opposed to hospital care, especially since the emphasis on research to date to document and improve antibiotic prescribing practices including ASPs has typically been on hospital care across Africa, including Tanzania and other LMICs [84,86,87,88,89,90,126]. This includes the strengthening of AMS in Tanzania under the Medicines, Technologies, and Pharmaceutical Services (mTaPS) program (2018–2023) (Table 1) [40,127].

### 2.3. Documentation Strategy and Suggestions for the Future

All documented studies will include the authors, the publication year, a summary of the aims and methodology, as well as the key findings. In addition, whether the ambulatory care setting included either public or private facilities or both will be acknowledged. This is because, as mentioned, we are aware that prescribing patterns may vary across the ambulatory care sectors in Tanzania [61,97].

Where possible, we will report antibiotic utilization according to their AWaRe classification: ‘Access’, ‘Watch’, or ‘Reserve’ [21,128]. The ‘Access’ group of antibiotics are considered as first- or second-line antibiotics for a range of common or severe clinical syndromes and typically have a narrow spectrum alongside low resistance potential. The resistance potential and side effects are higher among antibiotics in the ‘Watch’ group; consequently, their prescribing should be carefully considered among HCPs when prescribing these antibiotics. This is in line with recommendations in the AWaRe book [22,23,129]. The ‘Reserve’ group should rarely, if ever, be prescribed in ambulatory care; ideally, these antibiotics should only be prescribed as a last resort in hospitals [21,22,128,129]. The initial target for ‘Access’ antibiotics is 60% of total utilization across sectors; however, this is likely to vary across countries [22,25].

Finally, regarding possible future strategies among the key stakeholder groups, then, as mentioned, we will build on the considerable experience of the co-authors, similar to the situation in South Africa [110].

## 3. Results

The findings from the narrative review, along with suggested next steps, will be divided into sections. This is in line with the key questions outlined in the Methodology section.

The key components include the following:

- Antibiotic prescribing patterns among both public and private ambulatory care settings in Tanzania in recent years;
- Knowledge and attitudes towards antibiotics, AMR, and ASPs among all key stakeholder groups involved in prescribing practices in ambulatory care;
- Prescribing and quality indicators used in recent years in ambulatory care settings in Tanzania to improve prescribing practices;
- Details of any ASPs that have been implemented in ambulatory care settings in Tanzania and beyond in recent years to improve future prescribing practices of antibiotics and their impact, where known;
- Potential activities that can be undertaken by all key stakeholder groups in ambulatory care in Tanzania in the short to medium terms to improve future appropriateness of antibiotic prescribing practices, thereby helping to reduce AMR.

### 3.1. Prescription of Antibiotics in Public Ambulatory Care Facilities in Tanzania

Wiedenmayer et al. (2021), in their study among 120 public facilities, emphasized the importance of managing patients with infectious diseases appropriately in Tanzania with only a limited number of patients (1.2%) presenting with non-communicable diseases (NCDs) [116]. This may be why there has been appreciable prescribing of antibiotics in public ambulatory care facilities in Tanzania (Table 3) despite often limited evidence for their appropriateness [60,130,131,132,133,134]. However, in their study, Acam et al. (2023) ascertained that overall only 40% of patient encounters resulted in an antimicrobial being prescribed [78], which was lower than that among healthcare facilities in Ethiopia (63%), Sudan (62%), and Kenya (54%). Having said this, it is still appreciably higher than the WHO recommendation of 20% of encounters [78].

The high prescribing rates seen in practice in Tanzania may be influenced by fear or worse health outcomes if antibiotics are not prescribed; expectations from patients, especially if they have limited knowledge regarding antibiotics and AMR; as well as the potential influence from pharmaceutical company promotional activities [76,135].

Encouragingly, when antibiotics were prescribed, these were typically from the ‘Access’ list as opposed to the ‘Watch’ list, with little or no evidence of prescriptions antibiotics from the ‘Reserve’ list [131,132,134,136]. This is similar to the findings of Mbwasi et al. (2020), who found that >90% of all antimicrobial consumption in Tanzania, based on import data, was ‘Access’ as opposed to ‘Watch’ and ‘Reserve’ antibiotics [28].

**Table 3:** Public sector ambulatory care antibiotic utilization patterns.

Author, Year, and Setting	Objective/Aim and Methodology	Summary of Key Findings Including the Prescribing of Antibiotics via AWaRe Classification * Where Documented
Irunde et al., 2017 [61], exit interviews in randomly selected public and private facilities conducted in 2014	Cross-sectional study to assess the extent of the rational prescribing practice of medicines in four regions of Tanzania using the WHO/INRUD medicines use indicators The study used both retrospective and prospective data, with the extent of rational prescriptions assessed against WHO/INRUD criteria 2067 prescriptions were collected from 67 healthcare facilities in four studied regions	The average number of medicines per prescription was 2.3 67.7% of prescriptions contained an antibiotic 96.7% of medicines prescribed were in the national EML The Index of Rational Drug Prescribing was higher in public (3.65) versus private (3.02) facilities
Fink et al., (2020) [77], healthcare facilities in LMICs including Tanzania between May 2006 and December 2016	Cross-sectional study with the objective of improving the knowledge of antibiotic use in children under 5 seeking treatment in ambulatory care outpatient clinics in LMICs including Tanzania The study objectives were achieved by combining community-based data on treatment-seeking behavior with direct observation data on antibiotic during outpatient healthcare facility visits by children under 5 years of age	The mean number of antibiotics prescribed per sick child visiting a healthcare facility in Tanzania was 0.69—similar to the overall total among 8 participating LMICs including 6 from Africa and lower than that of Uganda, at 0.83 Overall, including Tanzania, the highest mean number of antibiotics prescribed to sick children who visited healthcare facilities was to children with ARIs (0.856), followed by children with diarrhea (0.55) and children with malaria (0.296)
Emgård et al., 2021 [130], primary HCWs’ experiences via in-depth interviews conducted in 2019	Qualitative study to ascertain 20 primary HCWs’ experiences regarding the prescribing practices of antibiotics for children under 5 years of age and their perceptions of AMR In-depth interviews with 20 primary HCWs	HCWs relied mainly on clinical examination and medical history to determine the need for antibiotics. However, confidence in giving advice concerning non-antibiotic treatments varied Some HCWs gave advice for symptomatic treatment after having ruled out specific diagnoses typically requiring antimicrobials However, other HCWs tended to prescribe antibiotics for children with fever on the basis of previous experiences enhanced by mothers’ expectations of a prescription for antibiotics
Huth et al., 2021 [131], Pediatric Outpatient Department in a regional referral hospital with patients recruited between August and December 2016	Outpatient-based cross-sectional study to ascertain any discrepancies between the prevalence of malaria infections in presenting children in the Lake Victoria Region and the extent of prescriptions for antibiotics and antimalarials Prospective study involving 133 febrile children	10.5% of presenting children were malaria-positive Despite this, 35.3% of children received a prescription for antimalarials, 63.9% were prescribed antibiotics, and 24.1% were prescribed both While only 11.3% of children reported any clinical signs for UTIs, 45.1% were given UTI as a working diagnosis and 73.3% were prescribed antibiotics 43.5% of children were prescribed one antibiotic, 27.1% were prescribed two antibiotics, and 29.4% received a combination of three antibiotics. Among the antibiotics, the most prescribed were ampiclox (FDC—−35%), gentamycin (25.6%—**A**), cephalosporins (21.4%), and ampicillin (12.8%—**A**) alone or in combination
Kilipamwambu et al., 2021 [132], PHC facilities between September 2018 and September 2019	Retrospective cross-sectional study conducted among 4 PHC facilities in Ilala district using WHO/INRUD core prescribing indicators The findings were used to suggest pertinent activities to improve prescribing practices where necessary	An average of 1.99 medicines were prescribed per consultation 51.9% of 1203 medicines prescribed (624) were antibiotics, with 97.6% on the current national essential medicines list Out of 624 prescriptions for antibiotics, amoxicillin was the most common (22.7%—**A**), followed by ciprofloxacin (13.6%—**W**) and metronidazole (11.6%—**A**) Penicillins were commonly prescribed for URTIs, nitroimidazoles were commonly prescribed for diarrhea, and fluoroquinolones were commonly prescribed for UTIs
Van de Maat et al., 2021 [133], PHC facilities between December 2014 and February 2016	Prospective study with the objectives of (a) providing insight into the case management of febrile children under 5 years in PHC facilities in Dar es Salaam; (b) identifying areas for improving the quality of care The study involved 547 febrile children aged 2–59 months treated in primary care facilities	Most diagnoses of presenting children were viral in origin: URTIs (60%) and/or gastro-enteritis (18%) 95% of presenting children (518/547) were prescribed antibiotics, with only 22% (119/547) having an indication for antibiotics based on local guidelines Antibiotic dosing was frequently out of recommended ranges Non-recommended treatments were common (29%) Vital signs, anthropometric measurements, and urinary testing typically failed to influence treatment decisions
Wiedenmayer et al., 2021 [116], 120 PHC facilities with the study conducted in 2012	Cross-sectional study undertaken in six districts within the Dodoma Region in Tanzania involving 120 randomly included facilities and 2872 patient cases The principal questions to asses in order to improve the future care of patients included (study aim): (i) Do prescribers comply with good prescribing practices? (ii) Do prescribers comply with current national STGs? and (iii) Are there any differences in adherence to STGs among facilities and for different disease areas?	The most prevalent conditions seen were URTIs (25%), malaria (18%), diarrhea (9.9%), pneumonia (6.1%), and skin problems (5.8%) Only 1.8% of all diagnoses among patients were for non-communicable diseases Only 29.9% of prescribers’ primary diagnoses completely adhered to current national STGs with 38.7% partially adhering, with non-adherence highest for skin problems and lowest for malaria The wrong medication was given in 30.9% of cases Overall, 61% of all patients were prescribed an antibiotic regardless of the diagnoses
Mabilika et al., 2022 [134], PHC facilities in 2 districts between January 2020 and December 2020	Retrospective cross-sectional study undertaken to ascertain current prevalence rates and predictors of antibiotic prescribing in primary healthcare facilities in the Dodoma region (study objective) This involved a retrospective review of the medical records following 1021 consultations 94.12% (961/1021) involved public primary healthcare facilities, with 5.88% among private and other faith-based facilities	Children < 5 years accounted for over 45% of the consultations An antibiotic was prescribed in 76.3% of consultations Amoxicillin (over 30%—**A**) and cotrimoxazole (over 29%—**A**) were the most prescribed antibiotics, with limited prescribing of ‘Watch’ antibiotics Over 98% (766/779) of antibiotics prescribed were on the Essential Medicines List; however, only 45% of antibiotic prescriptions adhered to the current STGs The prescription of antibiotics by clinical officers was almost 2.55 times higher than that among medical doctors Patients with pneumonia and URTIs were 15.9 and 2 times more likely to be prescribed antibiotics, respectively
Acam et al., (2023) [78], review of studies conducted in East Africa and published between 1993 and 2017	The primary objectives of the systematic review included (i) characterizing antimicrobial prescription patterns in East Africa including Tanzania; (ii) determining the proportion of patient encounters resulting in antimicrobial prescriptions; (iii) determining the magnitude of inappropriate antimicrobial use in East Africa	In Tanzania, overall, 40% of patient encounters was antimicrobial This compares with Ethiopia at 63%, Sudan at 62%, and Kenya at 54% However, appreciably higher than the WHO recommendation of 20%
Ekholuenetale et al., 2023 [74], demographic and health surveys—surveys conducted every 5 years between 2006 and 2021	Secondary cross-sectional statistical data was use to ascertain the prevalence of ARI amongst children under 5 years age in sub-Saharan Africa including Tanzania (study objectives) The study used secondary cross-sectional statistical data from the demographic and health surveys	High rate of antibiotic prescribing in young children with ARIs attending healthcare facilities in Tanzania at 61% of attending patients This compares with, for instance, Rwanda and Congo (58% each), Namibia (52.0%), and Gabon (50.0%) Similar rates of antibiotic prescribing for ARIs were seen in urban facilities (63.7%) versus rural areas in Tanzania (59.6%)
Keenan et al., 2023 [136], mixed-method study among 3 East African countries including Tanzania between February 2019 and September 2020	Mixed method approach using both quantitative and qualitative data to assess the socioeconomic, attitudinal, and contextual factors associated with treatment-seeking pathways for patients with UTI-like symptoms among 3 East African countries including Tanzania attending healthcare facilities (study objectives) In addition, how key pathway points intersect with antibiotic use was explored Quantitative data from 6827 adult outpatients presenting with UTIs symptoms in the 3 countries Qualitative in-depth interviews with a minority	86% of patients overall visited medical facilities as their first step in treating UTI-like symptoms Visiting private clinics initially was more common in Tanzania versus Uganda and Kenya Having health insurance was associated with 20% reduced odds of self-treating—this association was strongest in Tanzania Among patients with multi-step pathways, 48% of patients reported taking antibiotics at step one (seeking treatment) and 42% reported taking antibiotics at a second step (medicine already tried) Most antibiotics were taken after visits to medical facilities, with the most common antibiotics prescribed being amoxicillin (**A**) and ciprofloxacin (**W**)
Pinto Jimenez et al., 2023 [76], HCPs across Tanzania among six selected countries with data collected in 2018	Mixed-method study to quantitatively measure awareness of antibiotic resistance among HCPs from six lower-middle- and upper-middle-income countries (UMICs) in both human and animal health involving Tanzania Questionnaire-based study among 1091 participants including 726 human HCPs of which 126 from Tanzania	63.5% of human HCPs from Tanzania stated that their medical decisions on prescribing antibiotics were driven by fear/worse health outcomes (versus 62.5% in Ghana, and 75.3% of HCPs in Nigeria) Prescribing decisions were influenced by the following: ○ Lack of availability of local resistance data (only available to 31.7% of HCPs); ○ Exposure to company advertising (50.8% of HCPs); ○ Promotional activities from medical representative promotion (88.8%—sometimes/always)

NB: ARI = acute respiratory tract infection; * AWaRe = Access (**A**), Watch (**W**), and Reserve (**R**) antibiotics [21,128]; FDC = fixed-dose combination; HCP = healthcare professional; HCWs = healthcare workers; LMICs = low- and middle-income countries; PHCs = primary healthcare; URTIs = upper respiratory tract infections; UTIs = urinary tract infections; WHO = World Health Organization.

### 3.2. Prescription of Antibiotics in Private Ambulatory Care Facilities in Tanzania

Similar to physicians in the public sector, there have been concerns regarding the appropriateness of antibiotic prescribing practices among physicians working in the ambulatory care private sector in Tanzania.

Table 4 summarizes current prescribing practices among physicians working in the private ambulatory care sector. Similar to the situation among public facilities, there has been considerable prescription of antibiotics for self-limiting conditions including URTIs [136,137]. In addition, there has been a general overprovision of care, including unnecessary prescriptions of antibiotics [98,138].

Of equal concern is the high rate of prescribing of antibiotics from the ‘Watch’ list seen in some studies, which reached up to 33.3% of antibiotics prescribed in one study [98,139]. Having said this, Mbwasi et al. (2020) found that >90% of all antimicrobial consumption in Tanzania, based on import data, was ‘Access’ as opposed to ‘Watch’ and ‘Reserve’ antibiotics, with the most utilization in the private versus public sectors [28]. Encouragingly (Table 4), there appeared to be no prescriptions of antibiotics from the ‘Reserve’ list, which is similar to findings among public ambulatory care facilities in Tanzania (Table 3). In addition, this was similar to the findings of Mbwasi et al., with <1% of imported antibiotics being from the ‘Reserve’ list [28].

### 3.3. Knowledge and Attitudes Concerning Antibiotics and AMR among Key Stakeholder Groups Involved in Ambulatory Care in Tanzania

There have been concerns with current knowledge and attitudes regarding antibiotics among key stakeholder groups in Tanzania (Table 5). This includes healthcare students, who will become future prescribers and dispensers [141,142].

Issues and concerns identified among the 14 studies in this narrative review, which need to be addressed going forward, include concerns with knowledge and attitudes regarding antibiotics, AMR, and ASPs. This is not helped by currently variable training on antibiotics, AMR, and AMS among universities in Tanzania [76], as well as a lack of sources giving guidance on future prescribing practices. The recently launched AWaRe book giving treatment guidance on a range of infectious diseases found in ambulatory care should help in this regard going forward [22,23]. Future activities could also include upgrading the curricula for HCPs to better equip them with the necessary confidence and skills to improve prescribing across the sectors [75,142,143]. Universities should also upgrade their continuous professional development (CPD) activities to address concerns with the knowledge and practices of HCPs in ambulatory care settings across Tanzania. This is in line with the objectives in the updated NAP of Tanzania (2023 to 2028) [26] and will be part of future suggestions going forward.

**Table 5:** Knowledge, perceptions, and attitudes towards antibiotics, AMR, and ASPs among key stakeholder groups across settings in Tanzania.

Author, Year, and Setting	Aims/Objectives and Methodology	Key Findings
Mbwambo et al., 2017 [144], randomly selected community members	Cross-sectional study to assess knowledge and attitude towards antibiotics Questionnaire study among 292 randomly selected respondents	62.7% of respondents had good knowledge regarding antibiotics use, with 78.1% answering correctly that antibiotics cannot cure all infections. However, only 34.9% correctly answered that antibiotics cannot kill viruses Respondents who had no formal education or only primary and secondary education had lower odds of having good knowledge with respect to antibiotics versus those with higher education Those with good knowledge concerning antibiotics and their use had higher odds of having adequate attitudes towards the use of antibiotic
Lyimo et al., 2018 [145], HCPs in Northern Tanzania	Assess the knowledge, attitudes, and prescription practices of HCPs towards antibiotics Structured questionnaire design among 217 HCPs	51.2% strongly agreed that inappropriate prescribing of antibiotics puts patients at risk 51.6% stated that their decision to start antibiotic therapy was influenced by a patient’s clinical condition 50.7% stated that they were influenced to prescribed antibiotics by positive microbiological results in symptomatic patients 62.7% reported they had access to/used antibiotic therapy guidelines when prescribing antibiotics However, only 24.0% of HCPs had received regular training and education regarding antibiotic prescription practices The authors concluded that training and education is needed for HCPs to improve their prescribing of antibiotics
Mboya et al., 2020 [135], patients in Northern Tanzania	Assessed knowledge of appropriate antibiotic use among patients in Northern Tanzania Exit interviews among patients collecting medicines from drug outlets	Only 25% of patients had adequate knowledge about the use of antibiotics, with higher levels of education and having health insurance associated with greater levels of knowledge concerning antibiotics and their use Sore throat and influenza were considered by 62.5% and 46.1% of the patients, respectively, as diseases that can be treated with antibiotics
Simon et al., 2020 [68], parents and guardians of young children in Tanzania	Assessed the knowledge of 730 parents/guardians of children under five regarding their knowledge of appropriate use of antibiotics Part of a study assessing antibiotic purchasing patterns in this population	54.6% of parents/caregivers had a low level of knowledge regarding antibiotics 32.9% reported that they had stopped taking a full course of the antibiotic if their symptoms had improved Only 22.7% responded that their purchasing and use of antibiotics without a prescription/medical consultation could potentially enhance AMR
Emgård et al., 2021 [130], interviews with HCWs in Tanzania	Ascertained primary HCWs’ experiences regarding the prescription of antibiotics for children under 5 years of age and perceptions of AMR In-depth interviews with 20 primary HCWs	AMR was mainly perceived as a problem for individual patients who were misusing antibiotics Continuous education and external seminars were valued by HCWs in increasing their knowledge, helping address inappropriate management, and creating a sense of belonging Some HCWs requested more training on AMR, with others stating that previous training had positively impacted their prescribing practices
Frumence et al., 2021 [146], structured interviews with key personnel in Tanzania	This study was designed to explore and describe policy actors as well as human and animal health practitioners’ perceptions of AMR and ASPs in Tanzania 111 semi-structured interviews. Of these, 9 were policy actors, 25 were laboratory technologists/technicians from public and private primary healthcare facilities, 23 were in charge of health facilities, 8 were pharmaceutical assistants, and 19 were dispensers	Principal strengths in response to AMR in Tanzania included (i) improved multisectoral collaboration and coordination of AMR activities among key personnel in human health and animal sectors, (ii) existence of a political will to combat AMR, (iii) existence of public awareness campaigns regarding the appropriate use of antimicrobials, and (iv) existence of AMS activities However ongoing concerns with (i) insufficient public awareness on AMR, (ii) currently limited community engagement in AMR activities, and (iii) currently weak sectoral or departmental cooperation to reduce inappropriate use of antibiotics and currently inadequate human resources to effectively tackle AMR
Gabriel et al., 2021 [147], consumers in Ilala Municipality	Cross-sectional study assessing knowledge of the rational use of antibiotics among consumers 960 consumers were consecutively enrolled from outpatient pharmacies among both public and private hospitals in the Ilala Municipality	20.4% and 52.4% of surveyed consumers, respectively, demonstrated good knowledge regarding the rational use of antibiotics and potential conditions for treatment However, 70.6% stated that they stopped antibiotic use after dose completion, 53.6% would request the same antibiotic from an HCP if they believed that this helped treat a similar infection in the past, and 42.3% were willing to use/request the same antibiotic if a friend or family member had previously used this to treat similar signs and symptoms Surveyed consumers believed influenza (50.7%), sore throats (61.4%), and UTIs (60.5%) could be effectively treated with antibiotics Overall, the majority of consumers in this study had poor knowledge regarding the rational use of antibiotics. However, those with a high level of education and with health insurance had good knowledge regarding the rational use of antibiotics
Lubwama et al., 2021 [141], medical and pharmacy students	Evaluated the KAP of final-year medical and pharmacy students on antimicrobial use and AMR at three universities in East Africa including Tanzania using a cross-sectional survey with a self-administered questionnaire (178 students from Tanzania)	Only 20.2% of final-year students had good overall total knowledge of antibiotics in Tanzania vs. 72% in Uganda and 40% in Kenya Knowledge about AMR was higher among students in Uganda (67%) and Kenya (65%) versus Tanzania (44%) However, 85% agreed that the mechanism of resistance to beta-lactams in *K*. *Pneumoniae* is mainly enzymatic and 72% of students had a good attitude and perception of antibiotic use Of concern though is that 45.5% of students wrongly agreed that prescribing broad spectrum antibiotics is always better even if there are narrower spectrum antibiotics available that are effective for the given condition. In addition, 30.9% did not understand resistance mechanisms based on microbiology reports
Mutagonda et al., 2022 [148], parents/guardians of children	Determined the parents/guardians’ KAP as well as factors associated with inappropriate use of antibiotics among children in Tanzania Questionnaire design among 2802 parents/guardians attending pediatric clinics in referral hospitals	Overall, only 10.9% had good knowledge about antibiotics and 82.0% had poor practices regarding the appropriate use of antibiotics, e.g., only 20% felt that infectious diseases are becoming more difficult to treat with antibiotics, only 20% felt that antibiotics are not useful in treating diarrhea, and only 30% felt that antibiotics are not useful in treating influenza or coughs Having a university degree, good knowledge, and positive attitudes towards antibiotics were significantly associated with the appropriate use of antibiotics in children
Nkinda et al., 2022 [143], physicians and pharmacists	Ascertained the experiences of physicians and pharmacists on the implementation of ASPs among children among referral hospitals including ambulatory care 28 participants using a semi-structured interview guide	Most participants were not conversant with the meaning of AMS, while some reported having heard about the term but were unaware of any ongoing ASPs Participants viewed patients as contributors to poor antibiotic practices, stating that they influence physicians to prescribe antimicrobials of their choice. Alternatively, some patients do not attend health facilities due to the overall costs involved, opting to purchase them directly from community pharmacies/drug stores Increased education must be provided to HCPs to implement ASPs/reduce AMR
Nkinda et al., 2022 [75], prescribers and dispensers	To assess if the KAP of prescribers and dispensers could drive the irrational use of antibiotics among children in Tanzania Quantitative and qualitative approaches among 108 participants including interviews	81.5% of prescribers had good knowledge regarding the use of antibiotics in children. However, poor practices were observed in 70.4% of them Poor to moderate knowledge of antibiotics among prescribers were shown by 24% and 4%, respectively, agreeing/strongly agreeing that it was hard for them to choose the right antimicrobial Negative attitudes were shown by 7.4% and 7.4% agreeing/strongly agreeing that prescribing antibiotics while a patient does not require them does not contribute to AMR Encouragingly, 94.4% used guidelines as their main source of information. However, 51.9% stated that pharmaceutical companies were a leading source of information regarding antibiotics Prescribers felt that private health facilities were profit-oriented which may impact the outcomes of patients Future training on antibiotics and AMR would be crucial to enhance appropriateness and to reduce AMR
Ogunnigbo et al., 2022 [142], healthcare students	Evaluate the knowledge and attitudes of African healthcare students (including those from Tanzania) towards AMR and AMS activities using a specifically designed questionnaire	More than two thirds of students had teaching on prudent antibiotic use (70.4%), diagnosis of infections (74.3%), and antibiotic treatment (77.2%) However, almost half stated that they did not know of any source of information on antimicrobials (47.0%), and approx. 50% indicated that one of the challenges in obtaining up-to-date information on antimicrobials was a lack of resources (50.6%). 52.2% reported though that they had no prior knowledge of AMS; 42% had heard of AMS and 5.8% were unsure 73.5% believed that unnecessary use of antibiotics makes them ineffective; antibiotics are associated with side effects including diarrhea, colitis, and allergies (66.7%); and that any person treated with antibiotics is at increased risk of AMR (49.9%) Approx. 50% also believed that antibiotics are not effective against viruses (53.6%), colds, and influenza (42.4%). However, this was not the case in a minority of students More than 50% believed that more learning on AMR and AMS should be offered (56.1%) and 55.9% believed that an application that provides essential information about antimicrobials would be helpful for their learning
Pinto Jimenez et al., 2023 [76], HCPs including Tanzania	To quantitatively measure awareness of antibiotic resistance among HCPs from six lower-middle and upper-middle income countries in both human and animal health involving Tanzania Questionnaire-based study among 1091 participants including 726 human HCPs (126 from Tanzania)	Median scores regarding antibiotic resistance ranged from 54.6 among Tanzanian human HCPs (lowest) to 63.5 among Peruvian HCPs (highest) Overall, only 25.4% of human HCPs in Tanzania had attended specific training on AMR or AMS vs. 30.8% in Ghana and 35.3% in Nigeria In addition, 48.4% of human HCPs in Tanzania were more concerned about the level of hygiene and sanitation than antibiotic resistance, lower though than Ghana (60.6%) and Nigeria (63.5%) 99.4% of surveyed human HCPs in Tanzania would like further training regarding antibiotic resistance
Virhia et al., 2023 [149], HCPs in Tanzania	Explored HCPs’ motivation to engage in health matters and broader roles in community health; technical training; awareness, knowledge, and perceptions of infectious diseases and AMR; and practices contributing to AMR; as well as any constraints in their daily practices Involving 24 in-depth interviews and focus-group discussions	Variable knowledge surrounding bacteria, viruses, and the causes of common infections with several misperceptions, e.g., anthrax as a virus, malaria can be transmitted sexually, and women can acquire UTIs from stepping in unclean water or on soil contaminated with urine Having said this, most medical officers and nurses could articulate their understanding of antibiotics and AMR, with HCPs demonstrating their awareness of issues relating to AMR. However, AMR was not always understood in relation to infectious diseases A concern is that patients often do not take their antibiotics as prescribed and often stop when they begin to feel better Obstacles to improving antibiotic use include poor physical infrastructures, diagnostic capacity, staffing levels, and access to treatment

NB: AMR = antimicrobial resistance; AMS = antimicrobial stewardship; ASPs = antimicrobial stewardship programs; HCPs = healthcare professionals; HCWs = healthcare workers; KAP = knowledge, attitude, and practices.

### 3.4. Quality Indicators Currently Being Used in Ambulatory Care in Tanzania

A number of prescribing and quality indicators have been used in Tanzania to assess the quality of current antibiotic prescribing practices in ambulatory care across the sectors. These are contained in Table 6. Documented prescribing and quality indicators can be part of future ASPs in Tanzania, with for example adherence to guidelines increasingly seen as providing good quality care in Tanzania and beyond [34,133,134].

### 3.5. Antimicrobial Stewardship/Quality Improvement Programs in Ambulatory Care in Tanzania

Exemplars of ASPs undertaken to date in ambulatory care in Tanzania (Table 7), coupled with those from other LMICs (Supplementary Table S3), can provide directions to all key stakeholder groups as key personnel in Tanzania make progress in the NAP to reduce AMR. Such activities are enhanced by a greater emphasis on ASP activities in the updated NAP [26].

The studies by Rambaud-Althaus et al. (2017), Olaoye et al. (2020), and Ogunnigbo et al. (2022) are important as they discuss the introduction of guidelines on smartphones to improve future antibiotic prescribing practices as the digital environment in Tanzania is improved (Table 1) [35,36,42,142,151]. Alongside this, the government’s star rating facility quality assessment program was introduced for both public and private healthcare facilities (Table 1) [37,38,99]. Such developments are important to be able to monitor prescribing practices against agreed-upon indicators to provide ‘real time’ feedback to prescribers as opposed to irregular reviews of paper-based patient records. Providing real-time feedback on adherence to well-proven, well-communicated, and accepted treatment recommendations has worked well in Stockholm, Sweden, with the ‘Wise List’ [153,154]. Real-time feedback to prescribers, as well as monitoring of their performance compared with their colleagues, have resulted in continued high adherence to suggested treatments in practice over time in Stockholm, providing exemplars to others [153,155].

**Table 7:** Quality improvement programs including antimicrobial stewardship programs to improve antimicrobial prescribing in ambulatory care in Tanzania and their impact.

Author and Year	Setting and Activities	Key Findings Including their Impacts
Hopkins et al., 2017 [150]	To research the impact of using rapid diagnostic tests for malaria on subsequent prescriptions of antibiotics in children with acute febrile illnesses in Africa and Asia including Tanzania 522,480 children and adults were enrolled—8 cluster/individually randomized trials and one observational study	Antibiotics were prescribed to 127,052/238,797 (53%) patients in the control groups and 167,714/283 683 (59%) patients in the intervention groups Antibiotics were prescribed to 40% (35 505/89 719) of patients with a positive test result for malaria and to 69% (39 400/57 080) of those with a negative result In most settings, patients with negative test results had more prescriptions for antibiotics than those with positive results for all commonly used classes, e.g., penicillins, trimethoprim-sulfamethoxazole, tetracyclines, and metronidazole, i.e., typically ‘Access’ antibiotics
Keitel et al., 2017 [152]	Determined whether e-POCT was non-inferior in terms of clinical outcomes to IMCI (ALMANACH) when managing febrile illness in children under 5 years Compared the proportion of antibiotic prescriptions and severe adverse events (deaths and secondary hospitalizations) between the 2 arms Overall, 3169 patients took part (randomized between the two arms)	The absolute proportion of clinical failures was 2.3% in the ePOCT (37/1586) vs. 4.1% (65/1583) in the ALMANACH arm—overall, a 43% reduction in the relative risk of clinical failure when using e-POCT Proportion of severe adverse events was 0.6% in the e-POCT arm vs. 1.5% in the ALMANACH arm (RR 0.42, 95% CI 0.20, 0.87, *p* = 0.02) Proportion of antibiotic prescriptions was substantially lower with ePOCT—−11.5% compared to 29.7% (RR 0.39, 95% (CI 0.33, 0.45, *p* < 0.001) With e-POCT, the most common indication for antibiotics was severe disease—this was non-severe respiratory infections with the control algorithm (ALMANACH) The authors concluded that e-POCT has the potential to improve the clinical outcomes of children with febrile illnesses whilst reducing antibiotic use
Rambaud-Althaus et al., 2017 [151]	To assess whether smartphones with guidelines vs. paper support enhances the rational use of medicines among children Pilot cluster-randomized controlled study in Tanzania among 9 primary healthcare facilities—allocated to smartphones, paper-based algorithm, and controls Key outcome measures included proportion of children checked for danger signs to antibiotic prescribing rates	504 consultations—−166, 171, and 167 in the control, paper, and phone arms, respectively Smartphones vs. paper algorithms resulted in a significant increase in children checked for danger signs—−41% versus 74% (*p* = 0.04). Antibiotic prescriptions dropped from 70% in the control to 26% and 25%, respectively, in the paper and electronic arms Overall, mobile technology in low-income countries appears implementable and can improve performance Additional POCTs may be needed to enhance appropriate management
Keitel et al., 2019 [92]	Research the safety and efficacy of using C-reactive protein (CRP) to decide on antibiotics among febrile children at risk of pneumonia Controlled non-inferiority at 9 healthcare centers Primary outcome was clinical failure by day 7; secondary outcomes were antibiotic prescription (day 0) and secondary hospitalization or death by day 30	1726 children were included (intervention: 868, control: 858; 0.7% lost to follow-up) 2.9% (25/865) had clinical failure in the intervention arm at day 7 vs. 4.8% (41/854) in the control arm (risk difference, −1.9%) 2.3% of children in the intervention arm vs. 345 (40.4%) in the control arm received antibiotics (RR, 0.06 [95% CI, 0.04–0.09] Fewer secondary hospitalizations/deaths in the intervention arm: 0.5% vs. 1.5% (RR, 0.30 [95% CI, 0.10–0.93])
Olaoye et al., 2020 [42]	Highlighted the development and implementation of an app to support prudent antimicrobial prescriptions and improved AMS in healthcare facilities in Ghana, Tanzania, Uganda, and Zambia Ascertained from HCPs’ and patients’ attitudes the use of a smartphone to review guidelines before prescribing antibiotics	The most visited section of the app were the National Prescribing Guidelines—accounting for 66.1% of the total number of hits On a daily basis, mobile phones (28.9%) and printed posters (13.2%) were the most frequently used sources for information on antibiotics among HCPs, with the CwPAMS App mostly used by nurses and other health workers More than 50% of patients had a positive attitude to the use of smartphone apps by HCPs and that this increases the quality of healthcare offered as well as quickens access to healthcare Patients’ greatest concern was that the use of mobile apps may distract from healthcare provision
Hogendoorn et al., 2022 [91]	Tested whether an algorithm using clinical signs and host biomarkers can predict bacterial community-acquired pneumonia vs. viral/unknown pneumonia among patients with LRTIs in outpatient clinics, thereby reducing the prescription of antibiotics A classification and regression tree analysis (CRT) was performed 110 patients with LRTIs and no exclusion criteria from 4 clinics were included	A CRT analysis was performed with the algorithm forcing the respiratory rate to be the first splitting variable in order to reduce unnecessary laboratory analyses The proposed model had a specificity of 88% and a sensitivity of 88% Using this algorithm restricted the number of patients being prescribed antibiotics to 33/110 (30%) instead of the 55/110 (50%), i.e., a decrease of 40%
King et al., 2021 [156]	Using a standards-based approach adapted to low-resource setting (SafeCare) involving assessments, mentoring, and training to improve the quality of care and to subsequently assess its impact in faith-based and private for-profit facilities Outcome measures included HCW compliance with IPC practices and proportion of SPs who were managed in accordance with STGs 29,608 IPC indications in 5425 provider–patient interactions were observed	Intervention facilities had 4·4% (95% CI 0·9–7·7; *p* = 0.015) higher mean SafeCare standards assessment score at the end vs. control facilities but no evidence of differences in clinical quality between intervention and control groups at endline Compliance with IPC practices occurred in 56·9% of intervention facilities vs. 54·7% in control facilities (*p* = 0.071) Correct management occurred in 27·0% of SPs in the intervention group vs. 29·2% in the control group The lack of any effect on clinical quality could reflect the insufficient intervention intensity and links between structural quality and care processes as well as scarcity of resources for quality improvement
Ogunnigbo et al., 2022 [142]	Ascertained the potential use of a developed app to guide antimicrobial prescriptions/to serve as an educational tool for healthcare students in Africa including Tanzania Structured questionnaire among healthcare students including those from Tanzania	55.9% of students believed that an application that provides essential information about antimicrobials would be helpful for their learning 52.1% believed a medical information app offline and on the go will help make more informed decisions about antibiotic use

NB: HCP = healthcare professional; HCW = healthcare worker; LRTIs = lower respiratory tract infections; POCT = point-of-care testing; IPC = infection, prevention, and control; SP = simulated patient; STG = standard treatment guidelines.

### 3.6. Suggested Activities in the Short to Medium Terms to Reduce Inappropriate Prescription of Antibiotics in Ambulatory Care Settings in Tanzania

Table 8 provides a range of suggestions in the short to medium terms to help improve antibiotic prescribing practices in ambulatory care in both the public and private sectors in Tanzania. This builds on the findings in Table 1, Table 2, Table 3, Table 4, Table 5, Table 6 and Table 7 along with ongoing initiatives, which include the star rating scheme to improve the quality of ambulatory care as well as ongoing AMS educational activities [37,40,41]. In addition, activities are ongoing within the functioning multi-sectoral coordinating committee for AMR activities [31]. As a result, they help to reduce any negative impact of promotional activities among pharmaceutical companies [75,76], which has been a concern in other African countries [157,158,159].

## 4. Discussion

To the best of our knowledge, we believe this is the first study in Tanzania to comprehensively document and review all aspects of antibiotic prescribing practices in ambulatory care across the country, the objective being to lay the foundation for suggested activities to improve future prescribing practices and to reduce AMR. Key aspects of this narrative review included documenting antibiotic prescribing patterns across the sectors in recent years as well as key stakeholder knowledge and perceptions towards antibiotics, AMR, and ASPs. In addition, ongoing activities are needed to improve future antibiotic prescribing in ambulatory care in Tanzania. Proposed activities include building on current quality improvement programs including increased digitalization in ambulatory care, as well as the instigation of prescribing and quality indicators increasingly based on the AWaRe book [22,23]. Such activities can help grow the number of ASPs that have already been undertaken in Tanzania.

The findings in this study highlight considerable issues and concerns regarding the current prescribing practices of antibiotics across the sectors, which can include the excessive prescription of ‘Watch’ antibiotics in private care settings [74,116,134,137,139]. However, encouragingly, this is generally not the case in Tanzania, with import data showing that 83.1% of imported antibiotics in recent years are from the ‘Access’ group versus only 10.1% from the ‘Watch’ group and <0.01% from the ‘Reserve’ group [27]. Limiting the prescribing of ‘Watch’ antibiotics, as well as discouraging the prescribing of any ‘Reserve’ antibiotics in ambulatory care, are an important first step to reducing AMR [21,24]. We have seen high rates of ‘Watch’ antibiotics being dispensed or prescribed in ambulatory care in other LMICs, which needs to be avoided where possible in Tanzania alongside reducing any prescriptions of azithromycin where pertinent [73,174,175,176]. However, encouragingly, this was generally not the case in Tanzania (Table 3 and Table 4). Concerns though with current appreciable prescriptions of antibiotics in ambulatory care in Tanzania, especially for self-limiting conditions such as ARIs and UTIs, needs to be avoided where possible, which is similar to other African countries [110,177,178,179,180,181,182,183].

Key activities to improve future prescribing practices of antibiotics in ambulatory care in Tanzania include improving the evidence base, building on current knowledge (Table 3, Table 4 and Table 5). Alongside this, there are encouraging ongoing efforts to improve digitalization in Tanzania, building on current activities (Table 1), as well as to develop and introduce easy-to-use apps that can provide treatment guidance and improve routine data collection (Table 8). These efforts are essential for the Ministry of Health, as well as Health Insurers, to be able to rapidly monitor the appropriateness of any antibiotics prescribed against agreed-upon guidance, building on agreed-upon prescribing indicators (Table 6). As mentioned, agreed-upon indicators are likely to be increasingly based on the recently launched AWaRe book with its extensive treatment guidance [22,23]. This is because there are concerns with current adherence rates to STGs in Tanzania, which is similar to other African countries [110,178,180,184,185]. Adherence to guidelines will be increasingly seen as a more appropriate marker of the quality of antibiotic prescribing practices in ambulatory care versus current WHO/INRUD criteria [34,161]. Regular monitoring of adherence to agreed-upon guidelines in ‘real time’, with pertinent ASPs instigated where necessary, is essential to improve prescribing practices in this key sector. Without this, it will be difficult to achieve the agreed-upon goals for reducing AMR in the Tanzanian NAP.

There is also a need, alongside activities with prescribers, to improve the knowledge of all key stakeholders regarding antibiotics, AMR, AMS, and ASPs given current concerns (Table 5 and Table 8). However, for the activities to be effective in improving future prescribing and in reducing AMR, a number of coordinated activities and technologies need to be in place, building on the objectives of the recent NAP update [26]. In addition, ASP activities need to be regularly followed up, else their impact may not be sustained [186]. This is because we have seen in practice that disjointed activities often fail to achieve target objectives [64,187,188,189]. Coupled with this, single activities are generally not as effective as multiple activities to achieve target outcomes as part of an ASP [110,114,115,187]. In view of this, it is essential that all key stakeholder groups work together in Tanzania in a coordinated fashion, with prescribing habits regularly monitored and rapidly fed back. As mentioned, this has worked well in Stockholm, Sweden, with the development, communication, and active monitoring of prescribing against the evidence-based ‘Wise list’ of medicines resulting in high adherence rates in practice [153,154,155]. These proposals build on current digital plans and activities in the ambulatory sector in Tanzania to improve future antibiotic prescribing practices (Table 1) [26,35]. Potential digital activities include the instigation of easy-to-use apps to routinely collect and rapidly analyze antibiotic prescribing habits. Alongside this are improved diagnostic tools as well as greater sensitivity testing in ambulatory care in the future, which will be helpful when discussing the appropriate management of essentially self-limiting infections with patients as well as those with proven bacterial infections [101,190].

Ongoing ASPs in Africa, including Tanzania as well as other LMICs (Table 7 and Table S3), can act as exemplars for future ASPs in the ambulatory care settings. We are aware that there have been challenges undertaking ASPs in ambulatory care in LMICs due to personnel and resource challenges [79]; however, this is beginning to change with a number of ASPs now being undertaken in Tanzania and beyond (Table 7 and Table S3). This is essential to improve the appropriateness of future antibiotic prescribing practices in ambulatory care in Tanzania and across Africa if AMR rates are to be reduced in this high-priority region [2,16].

To date, patients and the activities of their organizations have often been a forgotten element in enhancing the appropriateness of future antibiotic prescribing practices across LMICs. However, their role in influencing prescribing practices is increasingly being recognized. Concerns with the current knowledge of patients regarding antibiotics, AMR, and ASPs (Table 5), need to be addressed going forward as part of future activities. As a result, they help to reduce inappropriate requests for antibiotics when seeing HCPs, especially for self-limiting conditions. To enhance the success of future activities with patients, it is essential that key organizations and patients are part of any future communication campaign, including any campaigns via social media (Table 8). This is because we have already seen the negative impact that misinformation surrounding COVID-19 had on medicine utilization patterns across Africa and beyond, which needs to be avoided [117,166,167,168]. Potential activities going forward to reduce AMR in Tanzania are included in Table 8, and we will continue to monitor the situation. Clear and readily accessible information about non-antibiotic management strategies, especially for self-limiting conditions such as ARIs, alongside treatment expectations and the potential risks of inappropriate antibiotic use, should be provided in a phraseology by HCPs and others that will be easily understood by patients to avoid future confusion [170].

We are aware of the limitations of this paper. These include the fact that we did not undertake a full systematic review for the reasons provided. To address this, we have included an appreciable number of published papers and other sources discussing the current situation regarding antibiotic prescribing patterns in ambulatory care across Tanzania, the rationale for the patterns seen, as well as potential activities among all key stakeholder groups moving forward. The latter has been achieved with considerable input from senior-level co-authors from Tanzania, across Africa and beyond. In addition, we concentrated only on ambulatory care and not hospital care for the reasons stated. Despite these limitations, we believe our findings and suggestions for the future are robust, providing guidance to all key stakeholder groups in Tanzania.

## 5. Conclusions

Overall, there are considerable concerns regarding current inappropriate prescribing practices of antibiotics in ambulatory care in Tanzania and the subsequent implications for increasing AMR. As a result, a number of targeted activities are essential among all key stakeholder groups to improve the situation (Table 8). Potential activities include a greater evidence base regarding current prescribing patterns across the sectors alongside possible ways to improve the monitoring of prescribing practices against agreed-upon indicators and guidelines.

Consequently, the development of pertinent and agreed-upon prescribing or quality indicators is an essential next step to improve future prescribing practices. These are likely to be increasingly based on the recently launched AWaRe book and subsequently included as part of planned ASPs. Future ASPs can build on existing examples in Tanzania as well as other LMICs (Table 7 and Table S3). Successful activities to improve future antibiotic prescribing will typically involve coordinated and comprehensive activities among all key stakeholder groups. Educating the community by engaging social media and expanding university curricula to incorporate knowledge of antibiotics and AMR is critical to meet future needs. In addition, there is an urgent need to rapidly address any critical misinformation regarding AMR, AMS, and ASPs. We will continue to monitor the situation given the current urgency and the need to reduce AMR in Tanzania as part of ongoing NAP activities.

## Figures and Tables

**Table 1:** Ongoing national initiatives in Tanzania to improve antibiotic prescribing and to reduce AMR.

Group	Activities and Achievements	References
Ministry of Health and others—NAP and Guidelines	Tanzania developed and introduced its NAP to reduce AMR in 2017. Frumence et al. (2021) documented several achievements to date following the introduction of the NAP. These included the establishment of a functioning multi-sectoral coordinating committee to help with AMR activities including a governance structure. Additional AMR surveillance sites have recently been introduced alongside educational and other activities encouraging greater awareness of AMR in the community, as well as greater availability of guidelines at health facilities, to improve future prescribing (availability and use of guidelines at health facilities is seen as a crucial first step to improve future prescribing practices). The national STGs were updated and now include the AWaRe classification for antibiotics. The findings of Frumence et al. (2021) contrast the findings of Sangeda et al. (2020). Sangeda et al. (2020) found that whilst there were ongoing AMR surveillance activities in Tanzania, coupled with the implementation of ASPs following the NAP, these activities were still at a low level.	[26,29,30,31,32,33,34]
Ministry of Health and others—ehealth strategy (2013–2018)/star rating system	The adoption of digital technologies and innovative solutions to help improve care delivery increased. This includes greater decentralization. A star rating system was introduced in primary care to improve the quality of care, with funds made available to improve care provision in the public system, especially in disadvantaged rural areas. However, Davis et al. (2022) found that there were still challenges with infrastructure, access, and available resources, including healthcare professionals (HCPs), to effectively tackle AMR in Tanzania.	[35,36,37,38,39]
Medicines, Technologies, and Pharmaceutical Services (MtaPS) program in hospitals	Educational and other programs were undertaken to strengthen ASPs in hospitals—a key element of the 2023 to 2028 NAPs.	[40,41]
Commonwealth Pharmacist Association	Apps have also been developed, including for Tanzania to improve awareness of AMS among HCPs. This includes documenting the indication and duration of the prescription of antimicrobials in patients’ notes to help improve the appropriateness of antibiotic selection.	[42]

NB: AMR = antimicrobial resistance; AMS = antimicrobial stewardship; ASPs = antimicrobial stewardship programs; AWaRe = Access, Watch, and Reserve antibiotics [21,22]; HCPs = healthcare professionals; NAP = national action plan; STGs = standard treatment guidelines.

**Table 2:** Antimicrobial resistance patterns in Tanzania in recent years.

Sector/Setting	Author, Year, and References	Key Findings
Multiple sites across Tanzania (10 studies)	Camara et al., 2023 [32]	*S. aureus* resistance to clindamycin ranged from 33.3% to 68.4%, erythromycin resistance ranged from 35.6% to 76.3%, cotrimoxazole resistance was 82.6%, and ampicillin resistance was 100%; however, there was low resistance to cefoxitin (27.3%), tetracycline (34.9%), cotrimoxazole (26.5%), and ceftriaxone (11.1%). *S. aureus* resistance to methicillin was 66.7% in 2018. *K. pneumoniae* was resistant to ampicillin (100%), cotrimoxazole (96.3%), ceftriaxone (95.7%), amoxicillin/clavulanate (94.6%), ceftazidime (90.9%), gentamycin (86.4%), and cefepime (75.6%). Versus other Gram-negative pathogens, *E. coli* was more resistant to ampicillin, amoxicillin-clavulanic acid, gentamycin, tetracycline, ciprofloxacin, amikacin, third-generation cephalosporins (ceftazidime and ceftriaxone), and cefepime. *P. aeruginosa* was resistant to cefepime (93.8%).
Hospital	Joachim et al., 2017 [43]	Most *S. aureus* isolates (95.5%) were resistant to penicillin. Resistance to gentamycin, ciprofloxacin, kanamycin, linezolid, and mupirocin was 14.6%, 11.2%, 11.2%, 3.4% and 1.1%, respectively.
	Mikomangwa et al., 2020 [44]	Resistance to clindamycin, cefepime, and meropenem from multiple specimens was 68.9%, 73.2%, and 8.5%, respectively. 68.4% of *S. aureus* isolates were resistant to clindamycin. 56.3%, 75.6%, 93.8%, and 100% of *E. coli*, *Klebsiella* spp., *P. aeruginosa*, and *Enterobacter cloacae*, respectively, were resistant to cefepime. *P. aeruginosa* (31.1%) and isolated *Klebsiella* spp. (8.5%) were resistant (and 25% and 6.4% respectively had intermediate susceptibility) to meropenem.
	Silago et al., 2020 [45]	Out of 91 isolates from patients admitted with osteomyelitis to a tertiary hospital, 85.1% of isolates were *S. aureus*, of which 28.6% were confirmed as MRSA strains.
	Mnyambwa et al., 2021 [46]	Isolates exhibited high resistance levels to commonly used antibiotics including ampicillin, amoxicillin-clavulanic acid, erythromycin, gentamicin, tetracycline, trimethoprim, and third-generation cephalosporins (ceftriaxone and ceftazidime), as well as reserved antibiotics (clindamycin and meropenem). *S. aureus* isolates were resistant to most of the antibiotics tested, with 66.7% classified as MRSA infections.
	Moremi et al., 2021 [47]	Of 285 patients investigated, 123 (43.2%) carried ESBL-GNB in their intestines. 5 of the 123 ESBL-positive patients were colonized with two different bacteria, making a total of 128 ESBL producing isolates. *E. coli* (n = 95, 74.2%) formed the majority of ESBL isolates. The proportion of CTX-M-1 group genes among ESBL isolates tested was 94.9%.
	Mloka et al., 2022 [48]	68.2% of Gram-negative bacteria were ESBL producers. 61.5% of the Gram-positive bacteria were identified as beta-lactamase producers. Cefuroxime was the least effective of the tested cephalosporins, exhibiting the largest MIC (18.47 ± 22.6 mg/mL) versus clavulanic acid alone (5.28 ± 8.0 mg/mL) and clavulanic acid-cefuroxime (5.0 ± 12.32 mg/mL). 78.2% of all isolates were sensitive to chloramphenicol.
Ambulatory care	Gidabayda et al., 2017 [49]	Isolates were taken for children with UTIs attending pediatric outpatients. Most common bacterial species isolated were *E. coli* (46.2%) and *Klebsiella pneumoniae* (30.8%). Both exhibited low susceptibility to ampicillin, co-trimoxazole, and clindamycin. However, they were susceptible to ceftazidime, ciprofloxacin, and nalidixic acid.
	Msanga et al., 2022 [50]	Among HIV-negative and HIV-positive children in the community: ○ 59.5% of *S. aureus* isolated were MRSA. ○ 47.2% of *Enterobacteriaceae* were resistant to third-generation cephalosporins, and 69.7% exhibited ESBL phenotypes. ○ The proportion of resistance to amoxicillin/clavulanic acid, gentamicin, and meropenem was significantly higher among HIV-positive than HIV-negative children.
	Schmeider et al., 2022 [51]	Among patients with UTIs attending hospital outpatients: ○ *E. coli* isolates showed high resistance to cotrimoxazole (76%), ampicillin (74%), piperacillin (74%), and fluoroquinolones (37%). ○ However, they showed widespread susceptibility to meropenem (100%), fosfomycin (98%), piperacillin/tazobactam (97%), and amoxicillin/clavulanic acid (82%).
	Silago et al., 2022 [52]	Among patients with UTIs attending hospital outpatients: ○ Resistance among *E. coli* ranged from 0.7% (meropenem) to 86.0% (ampicillin) and from 0.0% (meropenem) to 75.6% (ampicillin) in other *Enterobacterales*. ○ 45.4% (108) of *Enterobacterales* and 22.4% (35) of Gram-positive bacteria were MDR. ○ 33 MDR patterns were observed among Gram-negative bacteria, predominantly AMP-CIP-TCY (21.3%), and 10 MDR patterns were observed among Gram-positive bacteria—most commonly CIP-GEN-TCY (62.9%).
	Mlugu et al., 2023 [53]	Among patients with UTI attending hospital outpatients: ○ 51% of all isolated bacteria were multidrug-resistant. ○ All isolated bacteria demonstrated high resistance (>85%) to ampicillin and co-trimoxazole. ○ 51.5% of isolates were biofilm-forming *E. coli* and demonstrated relatively higher antibiotic resistance vs. non-biofilm forming bacteria (*p* < 0.05).

NB: ESBL-GNB = extended-spectrum beta-lactamases producing Gram-negative bacilli; MDR = multidrug-resistant; MIC = minimum inhibitory concentration; MRSA = methicillin-resistant *Staphylococcus aureus*; *P. aeruginosa* = *Pseudomonas aeruginosa*; UTI = urinary tract infection.

**Table 4:** Private sector ambulatory care antibiotic utilization patterns.

Author, Year, and Setting	Objectives and Methodology	Summary of Key Findings Including the Prescribing of Antibiotics Via AWaRe Classification * Where Documented
Irunde et al., 2017 [61], exit interviews in randomly selected public and private facilities conducted in 2014	Cross-sectional study to assess the extent of the rational prescription of medicines in four regions of Tanzania using the WHO/INRUD medicines use indicators The study used both retrospective and prospective data, with the extent of rational prescriptions assessed against WHO/INRUD criteria 2067 prescriptions were collected from 67 healthcare facilities in four studied regions	The average number of medicines per prescription was 2.3 67.7% of prescriptions contained an antibiotic 96.7% of medicines prescribed were in the national EML The Index of Rational Drug Prescribing was higher in public (3.65) versus private (3.02) facilities
Rogawski et al., 2017 [63], documented antibiotic use from mothers of children >2 years from 8 countries including Tanzania, study conducted between 2009 and 2019	Prospective study to describe the frequency and factors associated with the use of antibiotics in children aged 2 years or under (study aims) Estimated the proportion of episodes of bloody/non-bloody diarrhea and respiratory illnesses treated with antibiotics across 8 countries	262 children from Tanzania were part of the study 48.2% of children with non-bloody diarrhea were prescribed antibiotics and 73.8% of children with bloody diarrhea were prescribed antibiotics during the course of the study. Metronidazole (**A**) was the most common antibiotic prescribed, followed by the penicillins (**A**) 62.2% of children with non-specific respiratory tract infections were prescribed antibiotics 96.3% of children with acute lower respiratory tract infections were prescribed antibiotics Pencillins (**A**) were the most prescribed antibiotic for respiratory illnesses
Khalfan et al., 2021 [139], claims forms from insured patients, study conducted in September 2019	Cross-sectional study to determine the prevalence as well as to describe the pattern of antibiotic prescriptions among NHIF-insured patients at health facilities in Ilala Municipality (study objective) Retrospective analysis of claim forms of 993 NHIF-insured patients	357 (46.4%) patients received an antibiotic prescription. Of these, 19.9% received more than one antibiotic The most common antibiotic prescribed were co-amoxiclav (**A**—−17.1%), amoxicillin (**A**—−16.5%), ampicillin/cloxacillin FDC (14.8%), metronidazole (**A**—−10.6%), and ciprofloxacin (**W**—9.5%) 60.8% of prescribed antibiotics were in the ‘Access’ group, 33.3% were in the ‘Watch’ group, 17.4% were in the ‘not recommended’ group, and none were in the ‘Reserve’ group 92.2% of the antibiotics prescribed were in agreement with the current Tanzania STG recommendations
King et al., 2021 and 2023 [98,138], standardized patients (SPs) visiting 227 health facilities—May to June 2018	Prospective study to conceptualize and develop a framework to measure the overprovision of healthcare (economic waste, unnecessary use of antimicrobials, and clinical high risk of harm to individual patients)—comparing for-profit and not-for-profit private outpatient facilities among 18 of the 22 regions in Tanzania 909 visits completed with 4 SP cases presented: asthma, non-malarial febrile illness, TB, and URTIs	53% of 1995 medicines prescribed and 43% of 891 tests ordered were considered unnecessary Overall, 81% of SPs received unnecessary care 67% received care harmful to public health, e.g., unnecessary antibiotics or antimalarials 6% received clinically harmful care 13% of SPs were prescribed an antibiotic from the ‘Watch’ list Overprovision of healthcare was common and widespread in both for-profit and not-for-profit facilities. However, providers who made more effort were more likely to treat patients correctly
Khalfan et al., 2022 [140], insured patients—September 2018	Cross-sectional study to identify factors that influence antibiotic prescribing among insured patients (ambulatory care/in-patients)—study objectives 993 patients were included in the analysis	Overall, 46.4% of patients were prescribed an antibiotic—−65.4% of children compared with 45.2% of adults and 23.0% of older adults, greatest (77.0%) in those attending lower-care facilities Prevalence of being prescribed an antibiotic was approximately 4 times higher in patients with chronic rhinitis, nasopharyngitis, or pharyngitis, versus those with no such diagnosis
King et al., 2022 [137], informed/uninformed standardized patients (SPs) visiting 227 facilities—May to June 2018	Prospective study to understand key issues and concerns regarding the influence of patients and their knowledge on antibiotic prescribing practices for uncomplicated URTIs in clinics in the private sector in Tanzania—study objectives Randomization (1:1) of SPs with URTIs among 227 facilities with URTIs (‘I have a cough, and my head and throat hurts’)	Providers carried out an average of 6.35 of the recommended 20 history questions/physical examinations with informed SPs vs. 5.64 with uninformed standardized patients 89.9% of patients were prescribed an antibiotic (86.0% in the informed group and 94.8 in the uninformed group) 73.1% of antibiotics prescribed to SPs were penicillins (158/216)—of these 44.3% were ampicillin/cloxacillin FDC and 38.6% were amoxicillin (**A**) 11.5% of SPs were prescribed a ‘Watch’ antibiotic—greater in the informed group (14.9%) vs. uninformed group (7.96%)
Keenan et al., 2023 [136], mixed-method study among outpatients in 3 East African countries including Tanzania between February 2019 and September 2020	Mixed-method study to assess the socioeconomic, attitudinal, and contextual factors associated with treatment-seeking pathways for patients with UTI-like symptoms among 3 East African countries including Tanzania attending healthcare facilities In addition, explored how key pathway points intersect with antibiotic use using a mixed-method approach Quantitative data from 6827 adult outpatients presenting with UTIs symptoms in the 3 countries Qualitative in-depth interviews with a minority	86% of patients overall visited medical facilities as their first step in treating UTI-like symptoms Visiting private clinics initially was more common in Tanzania versus Uganda and Kenya Having health insurance was associated with 20% reduced odds of self-treating—this association was strongest in Tanzania Among patients with multi-step pathways, 48% of patients reported taking antibiotics at step one (seeking treatment) and 42% reported taking antibiotics at a second step (medicine already tried) Most antibiotics were taken after visits to medical facilities, with the most common antibiotics being amoxicillin (**A**) and ciprofloxacin (**W**)

NB: ARI = acute respiratory tract infection; * AWaRe = Access (**A**), Watch (**W**), and Reserve (**R**) antibiotics [21,128]; EML = Essential Medicines List; HCP = healthcare professional; HCWs = healthcare workers; LMICs = low- and middle-income countries; NHIF = National Health Insurance Fund; STG = standard treatment guidelines; TB = tuberculosis; URTIs = upper respiratory tract infections; UTIs = urinary tract infections; WHO = World Health Organization.

**Table 6:** Prescribing/quality indicators used to assess the quality of antimicrobial prescribing practices in Tanzania.

Indicator—Activity/Performance	References
Mean number of antibiotics prescribed per patient for a given diagnosis	[63,77]
% adherence to WHO/INRUD core prescribing indicators, including the number of encounters resulting in antibiotics being prescribed and whether antibiotics prescribed are on the current national EML	[61,78,132]
% of antibiotics prescribed adhering to current EML or STGs	[116,133,134,139]
% of non-recommended treatments prescribed	[133]
% of dosing of antibiotics within agreed ranges	[133]
% of patients prescribed the wrong medication	[116]
% of patients over-prescribed antimicrobials	[98,138]
% of patients prescribed antibiotics for infectious diseases including for respiratory tract infections/febrile illness (assessing over-prescribing)	[91,92,150,151,152]
% of patients prescribed ‘Watch’ antibiotics as opposed to ‘Access’ antibiotics	[98,137,139]
**Indicator—Outcome**	
% of clinical failures in children with febrile illness	[92,152]
% Secondary hospitalization or death by day 30 in children aged 2–59 months with fever and cough but without life-threatening conditions	[92]

NB: EML = Essential Medicine List; STG = standard treatment guidelines.

**Table 8:** Suggested activities in the short to medium terms to reduce inappropriate prescription of antibiotics in ambulatory care settings across Tanzania.

Key Groups	Suggested Activities
**Ministry of Health and health insurance groups**	**(A) Prescribers** Work closely with physicians and other HCPs/HCWs in both the public and private ambulatory care sectors to routinely monitor antibiotic prescribing practices as part of the national action plan to reduce AMR in Tanzania [26,29,31]. This is especially important given concerns with current prescribing patterns in ambulatory care (Table 3 and Table 4). Activities include gaining a better understanding of current utilization patterns and their rationale with the help of specially developed and easy-to-use electronic applications (apps) as well as introducing additional apps, alongside improved diagnostic aids, as well as encouraging greater culture and sensitivity testing in ambulatory care, to improve decision making [42,101,160]. This is part of digital technology programs being introduced in Tanzania to address ongoing concerns [35,160]. Alongside this, work closely with leading HCPs and their organizations to refine and agree on future quality/prescribing indicators to improve antibiotic prescribing practices and to move away from the use of WHO/INRUD criteria [161]. Future indicators are increasingly likely to be based on the AWaRe guidance following the recent launch of the book [22,23]. Any agreed-upon indicators can build on existing prescribing/quality indicators (Table 6). However, care is needed not to overload prescribers with too many indicators to monitor. Developments in digital technologies are essential to track current utilization patterns in ‘real time’ to regularly monitor prescribing against agreed-upon targets/indicators and to rapidly feedback the findings to improve utilization patterns in a reasonable time frame (difficult with paper-based systems). As part of these activities, prescribers should be made fully aware of the appropriate diagnostic codes they should enter. This may mean key Ministry of Health and Health Insurance groups instigating training sessions where pertinent, potentially surrounding the AWaRe book and guidance. Any training sessions are likely to involve hybrid learning building on the experiences during the COVID-19 pandemic across Africa [162]. Likely targets for ASPs in ambulatory care driven by Ministry of Health and Health Insurance groups include STGs to reduce unnecessary prescribing of antibiotics for essentially viral infections such as those with ARIs. The inclusion of the AWaRe classification of antibiotics in the updated Tanzania STGs should help in this regard [26]. Alongside these measures, Ministry of Health and Health Insurance group personnel should work closely with prescribers and other key stakeholders to reduce shortages of antibiotics, especially in the public sector, as this may adversely impact adherence to current STGs and AMR [163,164]. Multidisciplinary collaboration is also essential across sectors to improve future antibiotic prescribing practices as part of a one-health approach. The Ministry of Health/health insurance groups are a key element alongside HCPs and any patient advocacy groups to improve future antibiotic prescribing practices given increasing utilization in the past years [27]. However, this is changing with decreasing utilization in recent years [28]. **(B) Patients** Existing communication channels with patients should be improved given current knowledge concerns among patients (Table 5) as well as pressure on physicians and other HCPs from patients to inappropriately prescribe antibiotics. Inputs could be via targeted educational campaigns across the country including campaigns aimed at enhancing the appropriate management of essentially self-limiting viral infections (and limiting antibiotic use) via appropriate symptomatic treatment, with increasing use of social media especially following the pandemic to address misinformation and misconceptions [148,165,166,167,168,169]. It is essential though that patients understand the language being used by HCPs given concerns with scientific terminology including AMR and its meaning [170,171]. Such activities can be carried out alongside more traditional methods such as posters in HCP ambulatory care facilities and community pharmacies, as well as exploring alternative approaches to influence patients. This could include more personalized approaches given current knowledge concerns [172,173]. **(C) Universities** Ministry of Health and Health Insurance Groups should work with Universities to make sure future HCPs are fully aware of the core concepts surrounding prescribing and dispensing of antibiotics, AMS, and ASPs, given current concerns (Table 5). This can be achieved by Universities critically examining current curricula for undergraduate training alongside post-qualification educational inputs via continuous professional devel-opment (CPD) activities. Universities teaching health professions including medicine, pharmacy, nursing and other allied health professions should now routinely incorporate the rational use of antibiotics, including the WHO AWaRe classification and suggested treatments for a range of infectious diseases [22,23] into their curricula. Ministry of Health and Health Insurance Groups should routinely monitor this. Alongside this, Universities need to make sure that future HCPs are equipped during the course of their training with the necessary knowledge and skills for appropriate antibiotic prescribing in ambulatory care as well as promoting awareness regarding AMR and its causes, the objective being that HCPs are fully equipped and ready to start advising patients on appropriate treatments, especially for self-limiting conditions including URTIs, post qualification. All government/health insurance groups need to work with key personnel in universities to conduct research and monitor prescribing practices across sectors, including against agreed-upon indicators. This will be helped by ongoing programs to monitor the quality of care in ambulatory care facilities through increased digitalization [35,160]. This is essential to ensure ‘real-time’ feedback to accelerate change where pertinent. Following this, pertinent ASPs should be developed by Ministry of Health/ Health Insurance Groups with the help of University personnel and leading HCPs, and subsequently instigated, if targeted improvements in antimicrobial utilization in ambulatory care in Tanzania are not being reached. Potential ASPs can build on recent exemplars in Tanzania and beyond (Table 7 and Supplementary Table S3). **(D) Pharmaceutical Companies** Key personnel within government organizations and health insurance groups need to work with pharmaceutical companies to limit promotional activities that enhance the prescription of antibiotics, especially for self-limiting conditions as well as to minimize promotional activities aimed at enhancing the prescribing of ‘Watch’ versus ‘Access’ antibiotics. Alongside this, these key personnel should work with pharmaceutical companies to ensure that any promotional material is compliant with WHO recommendations given concerns in some African countries [157].
**Physicians and other associations dealing with ambulatory prescription across sectors**	Key physicians and their associations need to work closely with the Ministry of Health/health insurance groups and universities to design e-communication and other strategies to help limit patients’ expectations that they will be prescribed an antibiotic for essentially self-limiting conditions such as URTIs. This should also include effective ways to improve the education of patients to reduce inappropriate requests for antibiotics, especially for essentially self-limiting conditions. They also need to work closely with the Ministry of Health and others to link all health facilities together with coordinated IT systems and to put in place any necessary measures to promote cyber security to protect patients’ confidential data. They should provide input into ongoing CPD activities with universities and ambulatory care prescribers especially surrounding the AWaRe book and its implications, the objective being to optimize the care of patients with infectious diseases in ambulatory care whilst seeking to reduce AMR. Alongside this, key physicians and their associations need to work closely with Ministry of Health personnel/health insurance company personnel to introduce, test, and monitor pertinent prescribing/quality indicators, especially building on the AWaRe book and current indicators (Table 6) to improve the future prescription of antibiotics across the sectors—replacing the traditional WHO/INRUD indicators, which variably measure the quality of antibiotic prescribing [161]. As part of this, help introduce systems, building on current apps and digital technologies, that can better monitor antibiotic utilization in ambulatory care. Such activities are imperative as part of ongoing activities to improve appropriate prescribing practices in ambulatory care in Tanzania through agreed-upon prescribing/quality indicators. Key physicians and their associations also need to work with the Ministry of Health and others to reduce potential shortages of antibiotics, especially in public facilities—particularly if this means HCPs necessarily prescribing and dispensing ‘Watch’ vs. ‘Access’ antibiotics.
**Patients and patient associations, including advocacy groups**	Patients and their associations need to work with all key stakeholder groups to help ensure any messaging to patients in the form of social media campaigns, as well as more traditional approaches including posters, is understandable given the extent of misconceptions currently regarding antibiotics and their use, as well as AMR, in Tanzania (Table 5). Subsequently, work with key groups including government personnel to use social media appropriately to rapidly disseminate key messages regarding antibiotics and their use, as well as AMR, in a language that will be understandable to patients to address current misinformation. Such activities are especially important for essentially self-limiting conditions including ARIs. Work with key groups, including university groups, to enhance the communication skills of key prescribers when dealing with patients, especially surrounding infectious diseases that will be seen in ambulatory care settings in Tanzania. In addition, work closely with university groups to help dispel misconceptions surrounding antibiotics and AMR. Work with the Ministry of Health, health insurance groups, and others surrounding the development of pertinent future prescribing or quality indicators—especially with patients—a critical component to improve future appropriateness. These are increasingly likely to be based on the AWaRe book [22,23].

NB: ARI = acute respiratory illness; AMR = antimicrobial resistance; ASPs = antimicrobial stewardship programs; AWaRe classification [16,17]; CPD = continuous professional development; HCPs = healthcare professionals; HCWs = healthcare workers; URTI = upper respiratory tract infection.

## Data Availability

We have already referenced all sourced papers and publications.

## Supplementary Materials

The following supporting information can be downloaded at https://www.mdpi.com/article/10.3390/medicina59122195/s1, Table S1. International and national initiatives to reduce AMR; Table S2. Published studies assessing the extent of purchasing of antibiotics without a prescription in Tanzania; Table S3. ASP activities introduced among LMICs in recent years to improve antimicrobial prescribing in ambulatory care and their impact. These include additional references [191,192,193,194,195,196,197,198,199,200,201,202,203,204,205,206].
