# Supplementary material for: A Narrative Review of Recent Antibiotic Prescribing Practices in Ambulatory Care in Tanzania: Findings and Implications
Source: Medicina (Kaunas). 2023 Dec 18;59(12):2195. doi: 10.3390/medicina59122195 (PMC10745081; doi:10.3390/medicina59122195)
Supplement: Supplementary file 1 [file medicina-59-02195-s001.zip › medicina-2725817-supplementary.pdf]

Supplementary Material - Tables

Table S1. International and National Initiatives to reduce AMR

Organization	Activities	References
WHO alongside National Governments and Health Authorities	• Instigation of the WHO Global Action Plan to reduce AMR, translating into NAPs<br>• Key elements of NAPs include documentation of antibiotic prescribing and utilization patterns in humans as part of a One Health approach alongside ongoing and proposed activities to improve future antibiotic utilization in both human and animal health<br>• However, African countries are at different stages of the introduction and monitoring of their NAPs due to multiple challenges, which needs to be urgently addressed<br>• Challenges include limited resources and personnel, including champions, to drive forward agreed activities	[8-10, 17-20]
WHO alongside key members of the EML Group	• Reclassifying antibiotics into their AWaRe classification (Access, Watch and Reserve) with targets for use if Access antibiotics (initially 60% of total antibiotic use)<br>• Examples of antibiotics in the 'Watch' group include azithromycin, cefotaxime, ceftriaxone, ciprofloxacin, erythromycin, levofloxacin, meropenem, pipericillin, tazobactam and vancomycin<br>• Examples of antibiotics in the 'Reserve' group include colistin, fosomycin, linezolid and polymyxin<br>• Launch of the AWaRe book, which includes management suggestions for 26 common or severe clinical syndromes to reduce AMR . The AWaRe book, with its classification of antibiotics, takes into account the impact of different antibiotics and their resistance potential to reduce AMR.<br>• Antibiotics in the 'Watch' group should be carefully considered before prescribing whilst those in the 'Reserve' group should only be prescribed as a last resort in hospitals and prioritized for any Antimicrobial Stewardship Programme	[21-25]
African Society for Laboratory Medicine (ASLM), the African CDC, and the Southern Africa Centre for Infectious Disease Surveillance	Instigate initiatives to:<br>• Improve the surveillance of infectious disease and resistance patterns<br>• Develop and disseminate guidelines to treat common bacterial infections seen across Africa	[11-15]

NB: EML = Essential Medicines List; NAPs = National Action Plans; WHO = World Health Organization

Table S2. Published studies assessing the extent of purchasing of antibiotics without a prescription in Tanzania

**Author, year and reference**	**Key findings including the extent of purchasing of antibiotics without a prescription**
Horumpende et al, 2018 [65]	• Overall 92.3% of retailers visited by simulated patients dispensed antibiotics without a prescription<br>• The most commonly dispensed antibiotics were ampiclox for coughs and azithromycin for painful urination
Mboya et al., 2018 [191]	• The most prevalent form of irrational antibiotic use among patients who purchased antibiotics from community drug outlets was non-prescription usage - 76.3% of purchases were in this category<br>• Purchases of the incomplete doses and purchases of antibiotics for non-bacterial illness was widespread
Poyongo et al, 2020 [192]	Of the 197 surveyed pharmacists, 72.6% admitted to dispensing antibiotics without a prescription in their daily practice
Simon et al, 2020 [68]	• The proportion of parents purchasing antibiotics for under-fives was 47.7%<br>• The most commonly purchased antibiotic was amoxicillin (62.0% of purchases
Ndaki et al., 2021 [67]	• Dispensing of amoxicillin without a prescription was common (88.2%) among mystery clients at community drug outlets<br>• The majority of drug outlets typically only sold a half course of amoxicillin without a prescription - Mwanza (98%), Mbeya (99%) and Kilimanjaro (98%), with generally amoxicillin dispensed on demand when requested by clients without asking any questions
Mabilika et al., 2022 [193]	• The prevalence of purchasing of antibiotics without a prescription among surveyed households was 23.6% among rural respondents and 23.4% among urban respondents<br>• Self-purchasing was associated mostly with a perceived cough (76.3%/82%), body pain (71.1%/41.5%) and fever (63.2%/39.7%) among the different resident groups<br>• Amoxicillin was the most commonly dispensed antibiotic in both settings (47.3%/41%)
Ndaki et al, 2022 [66]	• 89.4% of drug sellers in community outlets recommended antibiotics to clients who described UTI like symptoms but had no prescription<br>• 58.93% were willing to sell less than the minimum recommended course.<br>• Community sellers recommended 32 different drugs to treat the same set of symptoms with only 7 currently appearing in the Tanzanian Standard Treatment Guidelines for UTIs
Ndaki et al, 2023 [69]	• In-depth interviews with 28 drug dispensers<br>• The majority of dispensers admitted to providing antibiotics without a prescription, selling incomplete courses of antibiotics and not providing detailed instructions to customers on how to use antibiotics
Olamijuwon et al., 2023 [118]	93% of community pharmacists and 97% of sellers in drug outlets did not ask the mystery clients for a prescription when requesting medicines to treat COVID-19 – typically antibiotics

Table S3. ASP activities introduced among LMICs in recent years to improve antimicrobial prescribing in ambulatory care and their impact.

Country, Author and Year	Objective and summary of the ASP intervention	Summary of the findings including the impact
China, Zhang et al., 2018 [194]	• The objective was to evaluate the cost-effectiveness of a health behaviour change intervention (information and leaflets were given to parents/caregivers) to reduce antibiotic prescribing<br>• The primary endpoint of the study was to lower the proportion of prescriptions containing at least one antibiotic given to children with URTIs as well as the cost-effectiveness associated with any reduction in the prescribing of antibiotics<br>• Costs included the average duration of consultations multiplied by the cost of the doctor's time combined with the cost of the leaflets, medicine and implementation costs	• There was an appreciable reduction (29%) in the prescribing of antibiotics in the active versus control arms (p = 0.0002)<br>• There was an incremental cost of $1.02 per patient in the intervention arm compared with the control arm<br>• However, when taking into account the cost of medicines and other costs, the incremental cost was $0.03/% point reduction in antibiotic use, which is close to cost-neutral<br>• Consequently, overall viewed as a success
China, Wei et al., 2019 [195]	• Initiatives involved principally education including multiple interventions to reduce outpatient prescribing of antibiotics<br>• Interventions comprised the following:<br>o Clinical guidelines<br>o Monthly prescribing review meetings<br>o Training in doctor–patient communication skills<br>o Provision of education materials for caregivers	• A 49% reduction in the prescribing of antibiotics for children with URTIs was seen after 6 months in the intervention arm—having adjusted for patient and prescribing doctor covariates<br>• The reductions persisted after 18 months but at a lower rate (-36%)<br>• Key factors for sustaining the reduction in antibiotic prescribing among physicians included their improved knowledge and communication skills combined with regular prescription review meetings
India, Dehn Lunn, 2018 [196]	• Principal education to reduce the rate of inappropriate prescribing for patients URTIs<br>• Multiple education activities consisted of a repeated process of audit and feedback combined with interactive training sessions, one-to-one case-based discussion, antibiotic guideline development and coding updates	• Antibiotic prescribing reduced from 62.6% of patients prescribed antibiotics for their URTIs to just 7.2% following the intervention<br>• This was combined with an increase in the documentation of examination findings from 52.7% to 95.6% of patients justifying the treatment approach

Kenya, Egger et al., 2017 [197]	• The objective was to improve the management of 4 common conditions in PHCs in Kenya: URTIs, UTIs, STIs and childhood diarrhoea through a comprehensive set of interventions<br>• Comprehensive and coordinated interventions included the following:<br>o Online educational programmes explaining the clinical guidelines for these 4 conditions<br>o Two-hour educational sessions<br>o Monthly feedback meetings<br>• Materials included posters and other materials to remind prescribers	• Adherence to agreed-upon clinical quality measures (CQM) increased from 41.4% to 77.1% for those PHCs that took part in the intervention<br>• However, adherence dropped slightly from 26.5% to 21.8% among the control PHCs over the 6-month study period. This was greatest for UTIs<br>• Adherence to agreed clinical quality measures significantly increased over the 6-month study period for the active intervention group<br>• Overall, the interventions were well received by those operating in the PHCs
Kenya, Korom et al., 2017 [198]	• Multiple interventions undertaken principally surrounding education to improve antibiotic prescribing in the ambulatory care setting. The interventions included the following:<br>• Formal introduction of a clinical practice guideline<br>• Introduction of peer-to-peer chart reviews<br>• Peer-reviewed publication describing local AMR patterns<br>• Interventions were undertaken by trained clinical officers	• Adherence to guideline-recommended antibiotics improved significantly, i.e., from 19% at baseline to 68% following the co-ordinated interventions (X2 = 150.7, $p < 0.001$)<br>• An outcome of composite quality scores improved significantly from an average of 2.16 to 3.00 on a 5-point scale (t = 6.58, $p < 0.001$)<br>• The interventions had different effects at different clinical sites—possibly reflecting differences in clinical officers and their activities<br>• Provider age was not a significant factor in subsequent changes in prescribing habits among participating HCPs in ambulatory care
Kenya, Kleczka et al., 2019 [199]	• Multiple components to this ASP among PHCs in the private sector in Kenya<br>• Interventions included the following:<br>o Rubber stamp templates for documenting the management of selected conditions, e.g., URTIs, UTIs, STIs and GIs<br>o Compilations of the relevant clinical practice guidelines for discussion/adherence<br>o One low-budget Android smartphone to each facility to facilitate data collection<br>o Six continuing medical education (CME) sessions at each facility every month for 6 months to improve compliance<br>• Adherence to guidelines was determined using two measures—these included the appropriateness of the diagnosis and the appropriateness of prescribing based on current guidelines	• Antibiotics were prescribed in 94.3% ± 1.6% of the 889 patient encounters documented with templates including 97.3% ± 2.3% for URTI encounters, 94.2% ± 3.8% for UTI encounters, 91.6%±1.1% for STI encounters and 91.3% ± 1.4% for GI encounters<br>• Overall template documentation scores (69.5% ± 1.7%) were significantly higher post the interventions<br>• The prescribing of nitrofurantoin (**A**) in patient encounters increased from 9.2% to 29.9%; $p < 0.0001$<br>• The prescribing of broad-spectrum quinolones (ciprofloxacin—**W;** norfloxacin—**W**) decreased from 30.0% of encounters to just 16.1% post intervention ($p < 0.05$)

Lao, Haenssgen et al., 2018 [171]	• The objective was to implement multifaceted educational activities in two Lao villages to share general antibiotic-related messages in order to reduce inappropriate antibiotic use<br>• Alongside this, learn about people's conceptions regarding antibiotics and AMR as well as health behaviours	• Overall there were mixed findings:<br>o The average recognition of the term for drug resistance rose from 27.6% to 91.4% among the educated group compared to 36.2% to 58.8% among the control group<br>o However, the impact of the educational activities on subsequent antibiotic use was inconclusive
Malaysia, Tay et al., 2019 [200]	• Activities were principally education involving educational toolkits.<br>• The toolkits included the following:<br>o The production of a training module for HCPs on ARIs and acute diarrhoea<br>o One-hour educational sessions covering diagnostic criteria and treatment decision pathways<br>o Educational posters in Malay and English in the waiting area and physician consultation rooms<br>o Multimedia educational videos in the waiting areas of healthcare facilities<br>o Physician reminders	• Overall, an appreciable reduction in antibiotic prescribing following the interventions:<br>o Prescribing of antibiotics for ARIs down from 29.1% to 13.7% of patients<br>o Prescribing of antibiotics for acute diarrhoea down from 11.2% to 6.7% of patients
Namibia, Brinkmann et al., 2020 [201]	• Activities included assessing the effectiveness of the implementation of ASPs among 10 PHCs in Namibia<br>• A SWOT analysis of each health facility was conducted by interviewing infection control focal personnel at each facility to assess their level of compliance with good AMS practices and policies	• 90% of the focal persons were aware of systems and policies for good AMS practices<br>• The level of compliance at hospital-based PHCs was 30.8% compared to clinics (9.1% to 36.4%)<br>• Principal challenges to implementing ASPs in Namibia among PHCs included:<br>o Lack of policies and systems specific to antimicrobial use hindered appropriate antibiotic prescribing<br>o There were also financial and human resource concerns with instigating ASPs
Sierra Leone, Hamilton et al., 2018 [186]	• Activities principally involved educational initiatives in this ASP in an outpatient department<br>• The ASP involved the provision of an empirical antimicrobial guideline introduced via a number of different methods. These included the following:<br>o One-to-one feedback meetings with prescribers<br>o Announcements of the guidelines in general meetings<br>Printed copies of the guideline in each outpatient room to help educate patients	• After the first cycle, the choice of the appropriate antimicrobial in prescriptions for the indication improved to 85%, and the correct antibiotic, dose and course length improved to 53%<br>• Unfortunately, after 2 months, the rates of appropriate prescribing reduced to 65% and 43%, respectively, following a lack of follow-up<br>• Overall, implementing guidelines can be effective in improving appropriate antibiotic prescribing in ambulatory care; however, repeated measures are needed for sustainable changes

South Africa, Blaauw and Lagarde, 2019 [202]	• Study using mystery patients being treated either by nurses in PHCs or by Private GPs for acute bronchitis to ascertain current prescribing practices<br>• In a follow-up study, mystery patients explicitly told GPs and nurses that they did not want antibiotics unless they were really necessary	• Antibiotic prescribing for acute bronchitis decreased by 20% compared with the first study when mystery patients requested not to be prescribed an antibiotic<br>• However, despite this, more than half of patients still received antibiotics even after stating they did not want one
South Africa, van Hecke et al., 2019, and Epps et al., 2021 [190,203]	• Explore the perceptions among 23 HCPs in publicly funded PHCs towards POCT<br>• Semi-structured questionnaires were used to document attitudes and experiences of existing POCTs as well as barriers and opportunities to introducing (hypothetical) new POCTs	• Largely positive experiences among HCPs with currently available POCTs<br>• HCPs were optimistic about the potential for new POCTs to support evidence-based prescribing decisions in order to reduce unnecessary antibiotic prescribing and to reduce the need for further investigations. In addition, support effective communication with patients, especially when antibiotics are unlikely to be beneficial<br>• However, resources, available space and workflow disruption are currently seen as barriers to their uptake into routine care
South Africa, De Vries et al., 2022 [204]	• Multidisciplinary audit and feedback meetings once a month at 13 PHCs to try and improve future antibiotic prescribing<br>• Ten antibiotic prescriptions were randomly selected for a peer review audit by the team, assessed and scored for adherence to seven key measures which included antibiotic choice measured against the current STG/EML<br>• All measures had to be met for a prescription to be considered correct<br>• Concurrently with this, primary care pharmacists monitored monthly consumption for the six oral antibiotics most prescribed, e.g., amoxicillin (**A**), co-amoxiclav (**A**), penicillin (**A**), azithromycin (**W**), ciprofloxacin (**W**) and flucloxacillin (**A**)—DDDs/100 prescriptions dispensed	• Mean overall level of adherence to guidelines following the interventions increased from 11% in July 2017 to 53% in June 2019<br>• However, prescribing adherence was significantly lower in the winter and spring, concurrent with higher antibiotic prescribing and consumption—this may reflect inappropriate antibiotic prescribing for increased viral ARIs during these months<br>• Mean of 19% correct prescriptions in the first 6 months (baseline) increased to a mean of 47% correct prescriptions in the last 6 months of the study following the interventions ($p < 0.001$)<br>• There was a reduction of 12.9 DDDs between the pre- and post-intervention periods ($p = 0.0084$), i.e., a 19.3% decrease in antibiotic consumption<br>• Overall, successfully demonstrating the favourable impact of this intervention
South Africa, Masetla et al., 2023 [205]	• The aim of this study was to provide AMS services to patients in a hospital's outpatient department with chronic bone and joint infections<br>• 44 patients participated, with questionnaires used to assess their understanding of their conditions as well as adherence to prescribed antibiotics	• 71 antibiotics were prescribed to these patients, with 62% from the 'Watch' group<br>• A total of 239 interventions were made, including educating patients and clinicians<br>• The majority of interventions regarding patients concerned their knowledge of their condition and the prescribed medication (61%)

	• Review of antibiotic prescriptions with prescribers contacted if concerns including their adherence to current STGs/EML	• 65 interventions (27%) were made regarding educating patients on adherence to prescribed antibiotics and their importance in helping resolve their condition<br>• The majority (96%) of the antibiotics were not prescribed according to the current STG; however, interventions were only needed in 31% of prescribed antibiotics since the STG only recommends empiric therapy directed against *Staphylococcus aureus*<br>• The majority of the drug treatment interventions concerned appropriate antibiotic selection (62%)
Vietnam, Hoa et al., 2017 [206]	• Multifaceted educational intervention over 7 months targeting the knowledge of HCPs towards antibiotics as well as their practical competencies and prescribing of antibiotics for patients with ARI in the intervention group<br>• There were no specific programmes in the control group	• Knowledge regarding antibiotics improved in the intervention group for patients with ARIs by 28%, antibiotic use for patients with mild ARIs by 15% and severe ARIs by 14%<br>• Practical competence for patients with mild ARIs improved in the intervention and control groups by 20% and 11%, respectively<br>• The practice regarding the prescribing of antibiotics for mild ARIs improved by 28% in the intervention group

NB: ARI = Acute Respiratory Illness; AMS = Antimicrobial Stewardship; ARIs – Acute respiratory illness; ASP = Antimicrobial Stewardship Programme; GI = Gastrointestinal infection; HCP = Healthcare Professional; PHC – Primary Healthcare; POCT = Point-of-care testing; STG – Standard Treatment Guidelines; STIs = Sexually Transmitted Infections; URTIs = Upper Respiratory Tract Infections; UTIs = Urinary Tract Infections
